# Trem2 Deletion Reduces Late-Stage Amyloid Plaque Accumulation, Elevates the Aβ42:Aβ40 Ratio, and Exacerbates Axonal Dystrophy and Dendritic Spine Loss in the PS2APP Alzheimer's Mouse Model

**DOI:** 10.1523/JNEUROSCI.1871-19.2019

**Published:** 2020-02-26

**Authors:** William J. Meilandt, Hai Ngu, Alvin Gogineni, Guita Lalehzadeh, Seung-Hye Lee, Karpagam Srinivasan, Jose Imperio, Tiffany Wu, Martin Weber, Agatha J. Kruse, Kimberly L. Stark, Pamela Chan, Mandy Kwong, Zora Modrusan, Brad A. Friedman, Justin Elstrott, Oded Foreman, Amy Easton, Morgan Sheng, David V. Hansen

**Affiliations:** ^1^Departments of Neuroscience,; ^2^Pathology,; ^3^Biomedical Imaging,; ^4^Bioinformatics,; ^5^Biochemical and Cellular Pharmacology, and; ^6^Molecular Biology, Genentech, Inc., South San Francisco, California 94080

**Keywords:** Alzheimer's disease, amyloid plaque, microglia, microgliosis, neuritic dystrophy, TREM2

## Abstract

*TREM2* is an Alzheimer's disease (AD) risk gene expressed in microglia. To study the role of *Trem2* in a mouse model of β-amyloidosis, we compared PS2APP transgenic mice versus PS2APP mice lacking *Trem2* (PS2APP;Trem2^ko^) at ages ranging from 4 to 22 months. Microgliosis was impaired in PS2APP;Trem2^ko^ mice, with *Trem2*-deficient microglia showing compromised expression of proliferation/Wnt-related genes and marked accumulation of ApoE. Plaque abundance was elevated in PS2APP;Trem2^ko^ females at 6–7 months; but by 12 or 19–22 months of age, it was notably diminished in female and male PS2APP;Trem2^ko^ mice, respectively. Across all ages, plaque morphology was more diffuse in PS2APP;Trem2^ko^ brains, and the Aβ42:Aβ40 ratio was elevated. The amount of soluble, fibrillar Aβ oligomers also increased in PS2APP;Trem2^ko^ hippocampi. Associated with these changes, axonal dystrophy was exacerbated from 6 to 7 months onward in PS2APP;Trem2^ko^ mice, notwithstanding the reduced plaque load at later ages. PS2APP;Trem2^ko^ mice also exhibited more dendritic spine loss around plaque and more neurofilament light chain in CSF. Thus, aggravated neuritic dystrophy is a more consistent outcome of *Trem2* deficiency than amyloid plaque load, suggesting that the microglial packing of Aβ into dense plaque is an important neuroprotective activity.

**SIGNIFICANCE STATEMENT** Genetic studies indicate that *TREM2* gene mutations confer increased Alzheimer's disease (AD) risk. We studied the effects of *Trem2* deletion in the PS2APP mouse AD model, in which overproduction of Aβ peptide leads to amyloid plaque formation and associated neuritic dystrophy. Interestingly, neuritic dystrophies were intensified in the brains of *Trem2*-deficient mice, despite these mice displaying reduced plaque accumulation at later ages (12–22 months). Microglial clustering around plaques was impaired, plaques were more diffuse, and the Aβ42:Aβ40 ratio and amount of soluble, fibrillar Aβ oligomers were elevated in *Trem2*-deficient brains. These results suggest that the Trem2-dependent compaction of Aβ into dense plaques is a protective microglial activity, limiting the exposure of neurons to toxic Aβ species.

## Abstract (precis)

*TREM2* is an Alzheimer's disease (AD) risk gene expressed in microglia. To study the role of *Trem2* in a mouse model of β-amyloidosis, we compared PS2APP transgenic mice versus PS2APP mice lacking *Trem2* (PS2APP;Trem2^ko^) at ages ranging from 4 to 22 months. Microgliosis was impaired in PS2APP;Trem2^ko^ mice, with *Trem2*-deficient microglia showing compromised expression of proliferation/Wnt-related genes and marked accumulation of ApoE.

## Introduction

Since the discovery of *TREM2* (triggering receptor expressed on myeloid cells 2) variants as genetic risk factors for Alzheimer's disease (AD) (Guerreiro et al., 2013; Jonsson et al., 2013), TREM2 biology has become a focal point in research efforts to better understand how the innate immune system impacts AD and other neurodegenerative diseases (Jay et al., 2017b; Ulrich et al., 2017; Yeh et al., 2017). However, whether Trem2 exerts protective or detrimental functions in mouse models of AD-related neuropathology has been rather unclear (Gratuze et al., 2018; Hansen et al., 2018; Ulland and Colonna, 2018).

In transgenic models of cerebral β-amyloidosis, plaque load has been reportedly increased, decreased, or unchanged in mice that lack Trem2, depending on the model, age, and brain region being analyzed (Jay et al., 2015, 2017a; Wang et al., 2015, 2016; Yuan et al., 2016; Parhizkar et al., 2019). *Trem2* deletion in amyloidosis models has also been reported to either increase or decrease phosphorylation of the endogenous tau protein (Jay et al., 2015; Wang et al., 2016). Similarly, studies in neurodegeneration models driven by transgenic expression of the human tau protein have suggested disparate roles of Trem2. In the hTau model (Andorfer et al., 2003), *Trem2* deletion increased the amounts of tau phosphorylation and aggregation detected (Bemiller et al., 2017). In contrast, in the PS19 model (Yoshiyama et al., 2007), *Trem2* deletion had a protective effect, preventing tau-driven synaptic loss and atrophy in the hippocampus and entorhinal cortex, respectively (Leyns et al., 2017).

Transcriptional profiling studies have defined a disease/damage-associated microglial (DAM) activation state that is commonly observed in the brains of neurodegeneration models (Deczkowska et al., 2018; Friedman et al., 2018). The acquisition of the DAM state is Trem2-dependent in mouse models of AD, amyotrophic lateral sclerosis, and demyelinating disease (Poliani et al., 2015; Wang et al., 2015; Keren-Shaul et al., 2017; Krasemann et al., 2017). Some have argued that this state of microglial activation or alarm is fundamentally protective (Keren-Shaul et al., 2017), whereas others have argued that this state is damaging and that returning microglia to their normal “homeostatic” state would be beneficial (Krasemann et al., 2017). It is conceivable that microglial TREM2 activity may be either protective or detrimental, depending on the disease stage and types of pathology present.

To clarify the role of Trem2 in β-amyloid-driven AD models, we studied the effects of *Trem2* deletion on microglial activation, plaque accumulation, and neuronal pathology in the PS2APP model across a wide range of ages and in both sexes. PS2APP mice develop amyloid plaque and attendant gliosis pathologies that increase with age, with female mice accumulating the pathology more rapidly than males (Ozmen et al., 2009). Here we report that the effects of *Trem2* deficiency on plaque load varied with age and sex, but notably, plaque accumulation was reduced at older ages in both female and male Trem2 KO (PS2APP;Trem2^ko^) mice compared with age-matched Trem2 WT (PS2APP;Trem2^wt^) mice. We consistently observed that the Aβ42:Aβ40 ratio was elevated, plaque morphology was more diffuse, and neuritic dystrophy histopathology was more marked in PS2APP;Trem2^ko^ mice, even at older ages when total plaque was reduced. Additional analyses of PS2APP;Trem2^ko^ mice at 12 months of age revealed ApoE-laden microglia, increased levels of soluble fibrillar oligomeric Aβ, and elevated neurofilament-L in the CSF. By RNAseq, we observed that proliferation-related transcripts in PS2APP;Trem2^ko^ microglia were reduced, particularly those encoding certain components and regulators of Wnt-related signaling. Collectively, our data indicate that Trem2-dependent microglial proliferation and activation attenuate the toxic effects of Aβ toward neurons (i.e., the DAM state is mainly protective) and that measurements of neuronal pathology are more informative than plaque load as readouts of microglial modulation in models of β-amyloidosis.

## Materials and Methods

### 

#### 

##### Animals.

All animal care and handling procedures were reviewed and approved by the Genentech Institutional Animal Care and Use Committee and were conducted in full compliance with regulatory statutes, Institutional Animal Care and Use Committee policies, and National Institutes of Health guidelines. Animals were housed in specific pathogen-free conditions with 12 h light/12 h dark/d and maintained on regular chow diets. The Trem2^tm1(KOMP)Vlcg^ null allele (C57BL/6N background) was crossed into the PS2APP model (C57BL/6J background). To generate experimental cohorts, all breeding mice were homozygous carriers of the PS2APP transgene and heterozygous carriers of the *Trem2*-null allele to allow maximal use of littermates between Trem2 WT (Trem2^wt^) and Trem2 KO (Trem2^ko^) PS2APP groups. We designed our study to analyze female cohorts at early (4 months), intermediate (6–7 months), and late (12 months) stages of pathology. We also analyzed males at the age of 6–7 months, a common age for us to examine PS2APP histopathology, to check whether any observed effects of *Trem2* deletion were sex-specific. The 4 and 6–7 month cohorts were processed together; the 12 and 19–22 month cohorts were separate batches. When we observed less plaque in 12 month PS2APP;Trem2^ko^ females, we decided to also analyze their still aging male counterparts to learn whether that effect was reproducible. (By that time, the numbers of aged males were somewhat depleted, so we included available Trem2^het^ mice to round out the analysis.) For dendritic spine analysis, animals also carrying the Thy1:GFP-M transgene (Jackson ImmunoResearch Laboratories, stock #007788) were used.

##### Flow cytometry and FACS.

Animals were anesthetized with ketamine/xylazine and transcardially perfused with 30 ml of ice-cold PBS before dissection of cortex + hippocampus. Care was taken to remove the choroid plexus and as much of the meninges as possible before dissociation and sorting. Tissues were dissociated and cell suspensions prepared as described previously (Srinivasan et al., 2016). All steps were performed on ice or at 4°C to prevent artifactual microglial activation.

**Figure 1.**
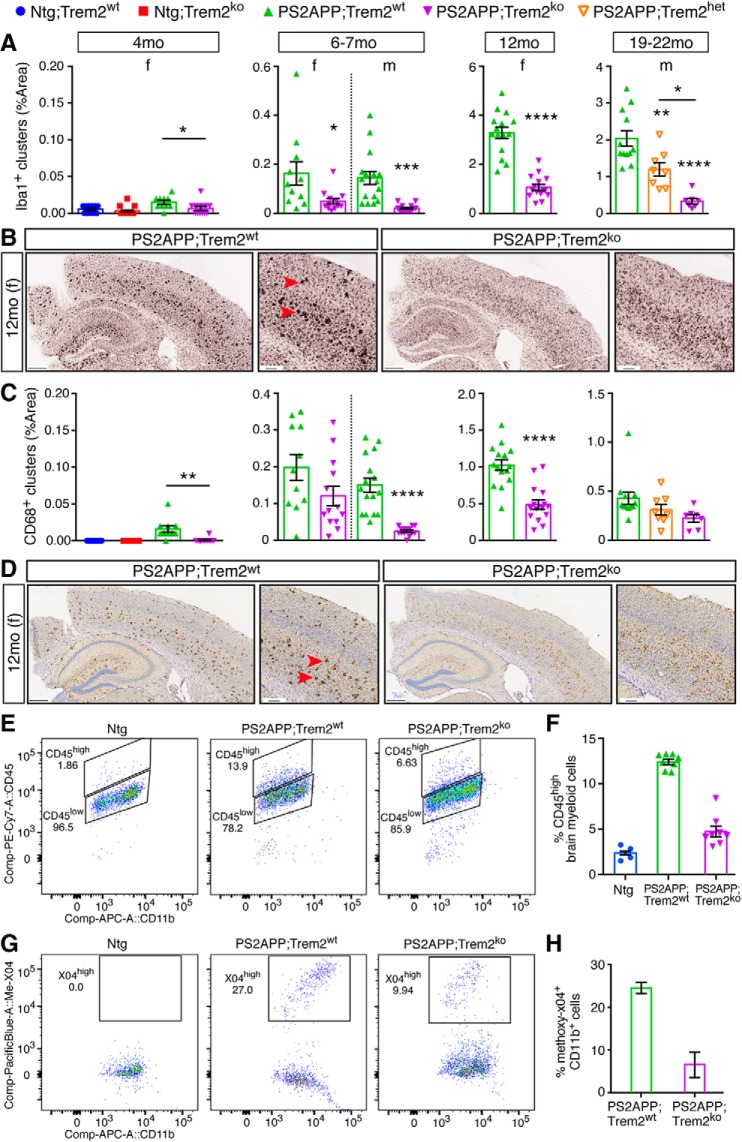
*Trem2* deletion impairs microglial clustering, activation, and plaque uptake in PS2APP β-amyloidosis model. ***A***, Immunohistochemical detection of microglial clustering in transgenic PS2APP mice or nontransgenic (Ntg) controls with WT (wt), heterozygous (het), or homozygous KO (ko) *Trem2* alleles. For analysis of total, rather than clustered, Iba1 signal, see Figure 1-1. Quantification of the percent area covered by clusters of Iba1^+^ microglia was measured from coronal sections of female mice at 4, 6–7, and 12 months of age, and from male mice at 6–7 and 19–22 months of age. Each data point represents the composite (average) histological score from several sections of an individual mouse. Bars and lines represent mean ± SEM. Unpaired *t* test for most cohorts, or by ANOVA followed by Tukey's multiple-comparison test for the 19–22 month cohort with three genotypes: *p < 0.05, **p < 0.01, ***p < 0.001, ****p < 0.0001 versus PS2APP;Trem2^wt^ or as indicated. ***B***, Representative low (left, scale bar, 400 μm) and high (right, scale bar, 100 μm) magnification images of Iba1^+^ staining in 12 month female mice are shown. Iba1^+^ clusters (red arrowheads) showed reduced presence across Trem2^ko^ mice at all ages. ***C***, ***D***, Same as in ***A*** and ***B***, except analyzing active microglial lysosomes as indicated by CD68 protein expression. ***E***, Representative flow cytometry plots measuring CD45 immunoreactivity (low or high) of CD11b^+^ brain-resident myeloid cells from 14 month PS2APP mice with or without Trem2 and from Ntg mice. ***F***, Percent of brain-resident myeloid cells with high CD45 expression measured from several mice of each genotype. Bars and lines represent mean ± SEM. ***G***, ***H***, Same as in ***E*** and ***F***, except analyzing plaque content in brain CD11b^+^ cells from ∼12 months mice injected with methoxy-X04 dye to stain amyloid material. Ntg mice are not plotted in ***H*** since they have zero methoxy labeling. *n* = 3 in Trem2^wt^; *n* = 2 in Trem2^ko^. For histological analysis of methoxy-X04 and Iba1^+^ microglia colocalization in sections, see Figure 1-1.

For quantifying CD45 immunoreactivity and collecting brain-resident myeloid cells by FACS, cell suspensions from 7 PS2APP;Trem2^wt^ (5 females, 2 males) and 7 PS2APP;Trem2^ko^ (6 females, 1 male) mice at 14–15 months of age were stained with the following antibodies in Hibernate-A medium for 20 min at 4°C on a rotator: APC-conjugated anti-CD11b (BD Biosciences, 561690, 1:200), PE/Cy7-conjugated anti-CD45 (BD Biosciences, 552848, 1:500), FITC-conjugated anti-Ly6g (Tonbo Biosciences, 35-5931, 1:200), and PE-conjugated anti-Ccr2 (R&D Systems, FAB5538P, 1:200). Samples were briefly washed and stained with DAPI before FACS sorting. Myeloid cells were selected by gating live (DAPI-negative) cells for CD11b and CD45 immunoreactivity. To avoid the presence of peripheral myeloid cells in the flow analysis and FACS collections, Ccr2^+^ cells (peripheral monocytes/macrophages) and Ly6g^+^ cells (neutrophils) were excluded. The total population of brain-resident myeloid cells (defined as CD11b^+^CD45^+^Ccr2^−^Ly6g^−^), consisting almost entirely of microglia but including CD45^high^ perivascular macrophages (∼2% of total), from each sample was collected in Hibernate-A. Collected cells were pelleted at 5000 rcf for 8 min, and RNA was extracted from cell pellets using QIAGEN RNeasy Micro kits. To measure CD45 immunoreactivity of brain myeloid populations during FACS, CD45^low^ and CD45^high^ gates were defined using a nontransgenic animal as a control to identify microglia in their normal state (CD45^low^). The same gates were transposed onto cells from PS2APP animals sorted on the same day in the same machine to ensure accuracy and consistency in determining percentages of CD45^low^ and CD45^high^ populations. Generally, animal pairs, including one PS2APP;Trem2^wt^ and one PS2APP;Trem2^ko^ animal, were processed together from perfusion through sorting. Data for nontransgenic animals in Figure 1*F* include the nontransgenic animals used to define the CD45 gates used during PS2APP cell sorting, as well as additional age-matched control animals from another cohort processed at another time (due to animal availability constraints).

For measuring microglial cells with β-amyloid content, animals at ∼12 months (*n* = 3 PS2APP;Trem2^wt^ and 2 PS2APP;Trem2^ko^) were intraperitoneally injected with methoxy-X04 (10 mg/kg) 24 h before tissue collection. Animals were processed as described above, and dissociated cells were stained for CD11b. Cells were also incubated with Calcein-AM (eBioscience, 65-0853-39, 1:1000) just before flow cytometry to label live cells using the 488 nm excitation channel. Live CD11b^+^ cells were gated as X04^+^ or X04^−^ using the DAPI excitation channel to determine the percentage of microglia with ingested amyloid content. Transgenic animals injected with PBS were also used as negative controls (data not shown).

##### RNA sequencing, differential expression, and gene set analysis.

RNA samples from sorted brain myeloid cells of 7 PS2APP;Trem2^wt^ (5 females, 2 males) and 6 PS2APP;Trem2^ko^ (5 females, 1 male) mice (14–15 months old) were selected for sequencing. The concentration of RNA samples was determined using DS-11 spectrophotometer (DeNovix), and the integrity of RNA was determined by 2100 Bioanalyzer (Agilent Technologies). Approximately 1–5 ng of total RNA was used as an input material for the library generation using SMART-seq v4 Ultra Low Input RNA kit (Clontech). Size of the libraries was confirmed using 4200 TapeStation and High Sensitivity D1K screen tape (Agilent Technologies), and their concentration was determined by qPCR-based method using KAPA Library Quantification Kit. The libraries were multiplexed and then sequenced on HiSeq4000 (Illumina) to generate 30 m of single-end 50 bp reads. Sorted cell RNA-Seq data were analyzed as described previously (Srinivasan et al., 2016). Briefly, Illumina adapters, low-quality sequences, and rRNA reads were first discarded. Remaining reads were aligned to the GRCm38 genome with GSNAP aligner (Wu et al., 2016), and reads overlapping each gene were quantified. Normalization was based on the nRPKM method, which is proportional to size factor normalization of DESeq (Love et al., 2014). Differential expression was performed using voom+limma (Law et al., 2014). Raw RNA-Seq data have been deposited to NCBI GEO under accession number GSE140744.

**Figure 2.**
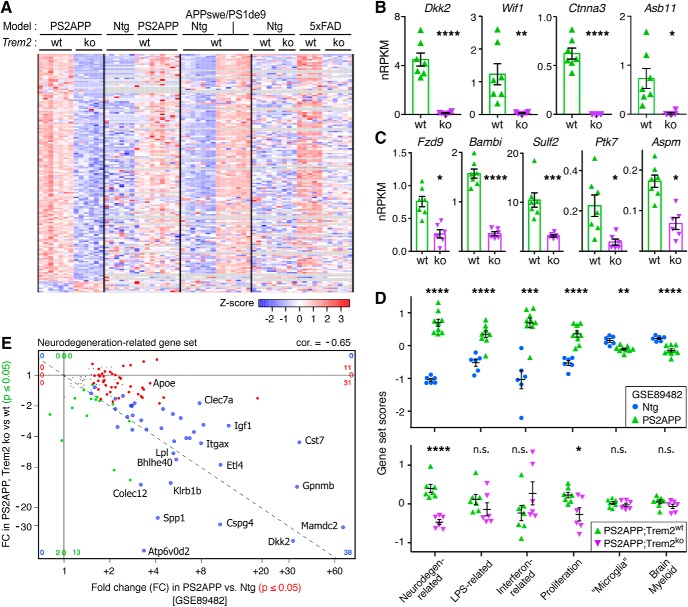
Induction of Neurodegeneration-related and Proliferation gene modules and Wnt-related signaling components is impaired in Trem2^ko^ microglia. ***A***, Heat map of differentially expressed genes (DEGs) between Trem2^wt^ and Trem2^ko^ microglia from 14 month PS2APP mouse brains (fold change ≥ 2, adjusted *p* ≤ 0.05), cross-referenced against previously published datasets from the PS2APP (GSE89482), APPswe/PS1de9 (GSE74615), and 5xFAD (GSE65067) models. Each row is a DEG, and each column is a microglial sort from a different mouse. *z*-score coloring represents a sample's distance in SDs from the mean expression value for a given gene across samples within a dataset. Most of the downregulated DEGs in Trem2^ko^ microglia were typically induced in models of β-amyloid pathology, and the majority also showed impaired microglial induction in Trem2^ko^ 5xFAD mice. For a complete list of genome-wide expression values in PS2APP;Trem2^wt^ and PS2APP;Trem2^ko^ microglia, see Figure 2-1). ***B***, Induction of the Wnt/proliferation regulators *Dkk2*, *Wif1*, *Ctnna3*, and *Asb11* was completely impaired in Trem2^ko^ microglia of PS2APP mice. ***C***, DEGs identified by GO analysis as “positive regulators of Wnt signaling” with reduced expression in PS2APP;Trem2^ko^ microglia included *Fzd9*, *Sulf2*, *Bambi*, *Ptk7*, *Aspm*, and *Dkk2*. ***B***, ***C***, Bars and lines represent mean ± SEM, with each data point representing microglial gene expression level from a given mouse. For genes in ***B*** and ***C***, see also Figure 2-2, showing microglial expression in the 5xFAD model with or without Trem2 (data from independent investigators). ***D***, Top, Analysis of previously published expression profiles from the PS2APP model (GSE89482) indicated that six gene expression modules for brain myeloid cells (defined by Friedman et al., 2018) showed altered expression in microglia from brains with β-amyloid pathology. Bottom, Analysis of Trem2^ko^ and Trem2^wt^ microglia expression profiles from PS2APP mice showed that the Neurodegeneration-related and Proliferation gene sets showed significant Trem2 dependence. For data from independent investigators showing enriched expression of Proliferation module in 5xFAD model microglia and its dependence on Trem2 for full induction, see also Figure 2-2). For heat map displays of all individual genes in each module, see also Figure 2-3. Each data point represents a gene set expression score for microglia isolated from a given mouse. Lines indicate mean ± SEM. Two-tailed *t* tests assuming unequal variance between groups: *p < 0.05, **p < 0.01, ***p < 0.001, ****p < 0.0001. ***E***, Four-way plot of Neurodegeneration-related gene set, with each point representing a gene's fold change in expression between PS2APP versus nontransgenic microglia on the *x* axis (red and blue points, adjusted *p* < 0.05) and between PS2APP;Trem2^ko^ versus PS2APP;Trem2^wt^ microglia on the *y* axis (green and blue points, adjusted *p* < 0.05). Blue points represent significant differential expression in both datasets. Tiny black points represent genes not differentially expressed in either dataset.

**Figure 3.**
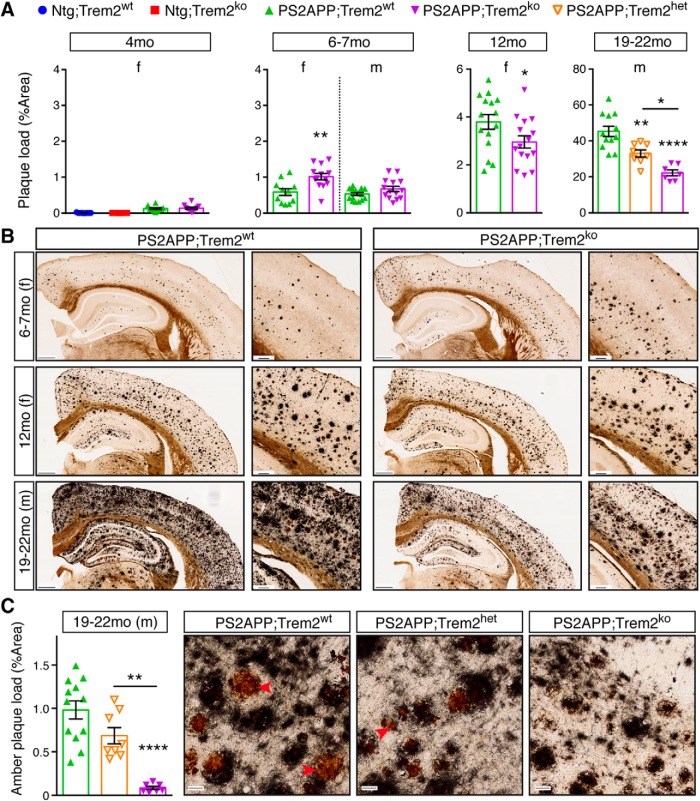
*Trem2* deletion shows age- and sex-dependent effects on amyloid plaque pathology, with reduced plaque accumulation at later ages. ***A***, ***B***, Amyloid plaque was visualized using the Campbell-Switzer silver stain method in nontransgenic (Ntg) controls or transgenic PS2APP mice with WT (wt), heterozygous (het), or homozygous KO (ko) *Trem2* alleles. Quantification of the percent area covered by amyloid plaque was measured from coronal sections of indicated sex and age. Representative low (left, scale bar, 400 μm) and high (right, scale bar, 100 μm) magnification images of amyloid stains are shown. In the absence of Trem2, plaque loads were elevated in 6–7 month females but reduced in 12 month females and in 19–22 month males. For two-photon imaging of methoxy-X04-labeled plaque in somatosensory cortex of 6 month females and 8 month males, see also Figure 3-1. ***C***, The Campbell-Switzer silver stain turns highly mature plaque cores amber (red arrowheads). Quantification plot (left) and representative images (right, scale bar, 20 μm) of amber core frequency in 19–22 month male mice. Error bars indicate mean ± SEM. Unpaired *t* test or ANOVA followed by Tukey's multiple-comparison test: *p < 0.05, **p < 0.01, ****p < 0.0001 versus PS2APP;Trem2^wt^ or as indicated.

Heat maps in Figure 2*A* and Fig. 2-3 were generated using gene sets of interest to compare transcriptional responses in multiple datasets. *z* scores were calculated as follows. First, log2-scale expression matrices were calculated as max(log2(nRPKM), −4). Then, each gene was centered and scaled to give *z* scores: for a given gene/sample combination, the *z* score represents distance of nRPKM value in SDs from the mean log2-scale expression value for that gene across all samples within a dataset. Rows (genes) were organized hierarchically using the Euclidean distance function. Columns (sorted microglia samples) were organized by project and genotype.

Gene ontology (GO) query was submitted on the PANTHER Classification System version 14.1 (Mi et al., 2019) at the website (http://pantherdb.org/) using the following inputs: Gene list = genes differentially expressed between Trem2^ko^ and Trem2^wt^ PS2APP microglia (fold change ≥ 2, adjusted *p* value ≤ 0.05); Organism = *Mus musculus*; Analysis = statistical overrepresentation test; Annotation set = GO biological process complete; Reference list = *Mus musculus* whole genome genes; Test type = Fisher's exact; Correction = Calculate false discovery rate (FDR).

For gene set enrichment analyses in Figure 2*D* and Fig. 2-2), each sample was assigned a gene set score using log_2_(nRPKM) values for each gene in the set. A sample's gene set score reflected the average difference, for all genes in the set, between that sample's measured log_2_(nRPKM) value for a given gene and the average log_2_(nRPKM) value for the same gene across all samples. In cases when one or more samples had no transcripts detected for a given gene, an imputed log_2_(nRPKM) value was assigned equal to one log_2_ step below the lowest log_2_(nRPKM) value detected for that gene in that sample set. We compared gene set scores between genotype groups using two-tailed *t* tests assuming unequal variance between groups.

##### Sectioning, histological and immunological staining.

Single-sex cohorts of animals used for histological and biochemical analyses included 4 month females (*n* = 11 nontransgenic Trem2^wt^, 14 nontransgenic Trem2^ko^, 10 PS2APP;Trem2^wt^, and 12 PS2APP;Trem2^ko^), 6–7 month females (*n* = 11 PS2APP;Trem2^wt^ and 13 PS2APP;Trem2^ko^), 6–7 month males (*n* = 16 PS2APP;Trem2^wt^ and 14 PS2APP;Trem2^ko^), 12 month females (*n* = 15 PS2APP;Trem2^wt^ and 15 PS2APP;Trem2^ko^), and 19–22 month males (*n* = 12 PS2APP;Trem2^wt^, 8 PS2APP;Trem2^het^, and 7 PS2APP;Trem2^ko^). Animals were deeply anesthetized with 2.5% tribromoethanol (0.5 ml/25 g body weight) and transcardially perfused with PBS. One brain hemisphere was drop-fixed in 4% PFA for 2 d at 4°C with agitation and then transferred to PBS for histopathological analyses. The other hemisphere was subdissected into cortical and hippocampal tissues that were frozen and stored at −80°C for biochemical assays. Immersion-fixed hemi-brains were cryoprotected, embedded up to 40 per block in a solid matrix, and coronally sectioned at 35 μm (MultiBrain processing by NeuroScience Associates) as previously described (Wang et al., 2011; Kallop et al., 2014). Sheets of sections were stored in cryoprotectant (30% glycerol, 30% ethylene glycol in PBS) at −20°C until use.

Immunohistochemical (IHC) stains for Iba1, CD68, and Gfap were performed at NeuroScience Associates as described previously (Wang et al., 2011), and CD68-stained sections were counterstained with Nissl (0.05% thionine/0.08 m acetate buffer, pH 4.5). Silver stains for amyloid plaque (Campbell-Switzer stain) (Switzer et al., 1993) and neuronal damage/degeneration (amino cupric silver) or “disintegrative degeneration” stain (de Olmos et al., 1994) were also performed at NeuroScience Associates. The bases for these silver stains are given by Switzer (2000) and described at the NeuroScience Associates website (https://www.neuroscienceassociates.com/technologies/staining/). IHC and silver stains spanned a broad rostral-caudal range, including 8–11 sections per animal. Stained slides were returned to Genentech for imaging and quantitation, and unused sections were also returned to Genentech for cryoprotected storage until used for additional stains.

For X-34 stains, sheets were mounted onto slides and completely dried. Slides were incubated with 10 μm X-34 in PBS containing 40% ethanol and 0.02 N NaOH for 10 min, followed by 3 quick washes in PBS, differentiation in 80% ethanol for 1 min, and additional 3 quick PBS washes. After applying ProLong Diamond Antifade Mountant (Thermo Fisher Scientific, P36961), slides were covered with no. 1 coverslips. Two sections per animal were stained, with all cohorts stained and analyzed simultaneously.

For costaining of plaque, microglia, and ApoE or dystrophic axons, sheets encompassing 2 or 3 sections per animal containing regions of the rostral and caudal hippocampus were washed in PBS and then PBS plus Triton X-100 (PBST, 0.1%) and then blocked in PBST (0.3%) with 5% BSA and 5% normal donkey serum, then incubated overnight with primary antibodies diluted in PBST (0.3%) plus 1% BSA at 4°C. Microglia were labeled with rabbit anti-Iba1 (Wako, 019-19741, 1:000) or goat anti-Iba1 (Abcam, ab5076, 1:1000), ApoE with a rabbit monoclonal antibody (Abcam, ab183597, 1:4000), and dystrophic neurites with rat anti-Lamp1 (Abcam, ab25245, 1:2000). Primary antibody incubation was followed by three 10 min washes in PBST, followed by incubation with secondary antibodies for 2 h at room temperature. Donkey anti-rabbit IgG-Alexa555, anti-rat IgG-Alexa647, and anti-goat IgG-Alexa647 (Thermo Fisher Scientific, 1:500) were used as secondary detection reagents. Following the stain, tissue sheets went through three 10 min washes in PBST (0.1%) and three quick washes in PBS. Sheets were mounted onto slides with 0.1% gelatin in PBS and allowed to dry and adhere to the slide at room temperature. To label plaque, slides were then incubated with 10 μm methoxy-X04 in 40% ethanol in PBS for 10 min, washed briefly in PBS, differentiated in 0.2% NaOH in 80% ethanol for 2 min, washed, and then allowed to dry. Slides were coverslipped with added ProLong Gold Antifade Mountant (Thermo Fisher Scientific, P36961). All cohorts were stained and analyzed simultaneously.

##### Imaging and quantitation of stained sections.

Brain tissue samples processed by NeuroScience Associates were imaged on the SCN400 whole-slide scanning system (Leica Microsystems) at 200× magnification. MATLAB (The MathWorks) running on a high-performance computing cluster was used for all whole-slide image analyses performed in a blinded manner. Quantification of CD68 or Iba1 staining and enlarged dark cluster areas was performed using morphometric-based methods as previously described (Le Pichon et al., 2013; Kallop et al., 2014). The large dark “cluster” of CD68 or Iba1^+^ cells coincided with the presence of amyloid plaques. Analysis of amino cupric staining was performed using color thresholds and morphological operations. Plaque area was analyzed from slides stained using the Campbell-Switzer method with plaques appearing with a black or amber hue. Multiple color classifiers spanning narrow ranges in RGB and HSV space were created for positive and negative features. Plaques were segmented using these classifiers and applying adaptive thresholding, Euclidean distance transform, morphological operations, and reconstruction. The percentage plaque load, amino cupric, Iba1, CD68, or Gfap positivity for the entire section were calculated by normalizing the positive pixel area to tissue section area and averaged from 8–11 sections/animal. All images, segmentation overlays, and data were reviewed by a pathologist.

Image acquisition of immunofluorescent slides costained for plaque, microglia, and either ApoE or dystrophic neurites was performed at 200× magnification using the Nanozoomer S60 or XR (Hamamatsu) digital whole-slide scanner. Ideal exposure for each channel was determined based on samples with the brightest intensity and set for the whole set of slides to run as a batch. Total tissue area was detected by thresholding on the Iba1 signal and merging and processing of the binary masks by morphological operations. Methoxy-X04, Lamp1, ApoE, and Iba1 staining was analyzed using a top-hat filter and local threshold followed by morphological opening and closing. For Lamp1 and methoxy-X04 staining, shape factor, roundness, and solidity features were used to eliminate elongated objects. In addition, a minimum size of 34 μm^2^ was applied to exclude small areas of staining. The detected plaques were used as markers in a marker-controlled watershed segmentation to create watershed lines of separation. The plaque mask was then dilated by 17 μm but constrained to be within watershed lines to prevent merging of plaques in close proximity during dilation. Total Lamp1-positive staining was normalized to the whole tissue area. Plaque-associated Lamp1 and Iba1 staining was constrained to be within the mask of dilated area around plaque and was normalized to the same area. Plaque-associated ApoE staining was constrained to be within the mask of plaque + dilated area and normalized to plaque area. Data were averaged from 2 or 3 sections per animal.

For X-34 stains, images were collected with a confocal laser scanning microscope LSM780 (Carl Zeiss) using Zen 2.3 SP1 software (Carl Zeiss). Eleven *z*-stack images at 1 μm intervals were collected with Plan-Apochromat 20×/0.8 M27, and maximum intensity projection images were created using Zen software. Images were collected and processed blind to genotypes. Image analysis was performed using MATLAB in a blinded fashion on the maximum intensity projection of the confocal *z*-stack. Control images that did not have X-34 positive staining were used to determine an initial threshold to exclude background. A threshold that is >99.99% of all pixel intensities in the control images was applied to all images to determine an initial segmentation mask. The binary masks were then smoothed out using morphological opening and closing. A minimum size of 9 μm^2^ was applied to exclude small areas of staining. A threshold corresponding to the 80th and 50th intensity percentile for the pixels within the segmentation mask of all positive images was applied to analyze compact and diffuse area, respectively. Post-threshold morphological operations and size exclusion were performed as described above. The plaque diffuseness index was calculated as follows: (Area_diffuse+compact_ − Area_compact_)/Area_diffuse+compact._ For each animal, data were averaged from two sections per animal, with 3 or 4 images per section consisting of two fields from cortex: one field from dorsal subiculum and/or one field from dentate gyrus molecular layer.

For analysis of methoxy-X04 or ApoE colocalization within Iba1^+^ microglia, images were collected from the costained slides described above for the 12 month female cohort of animals using confocal laser scanning microscope LSM780 (Carl Zeiss) with Zen 2.3 SP1 software (Carl Zeiss). Eleven *z*-stack images at 1 μm intervals were collected from the cortex with Plan-Apochromat 20×/0.8 M27. To determine colocalization of ApoE and Iba1 staining, or methoxy-X04 and Iba1 staining, we calculated the Mander's colocalization coefficients using the ImageJ plugin JACoP, as described previously (Dunn et al., 2011). The same thresholds were consistently used to identify the Iba1, ApoE, or methoxy-X04 channel across animal samples. Calculations were performed on the entire *z*-stack of images.

##### Aβ peptide measurements.

Frozen hippocampal tissues, described above, were homogenized in 10 volumes of TBS (50 mm Tris pH 7.5, 150 mm NaCl) including complete EDTA-free protease inhibitor mixture (Roche Diagnostics) with aprotinin (20 μg/ml) and leupeptin (10 μg/ml) in a QIAGEN TissueLyser II (3 min at 30 Hz). Samples were then centrifuged at 20,000 × *g* for 20 min at 4°C. Supernatants were collected as the “TBS fraction” and stored at −80°C until analyzed. The pellet was then homogenized in 10 volumes of 5 m guanidine HCl using the TissueLyser II and then placed on a rotisserie for 3 h at room temperature. Samples were diluted 1:10 in a casein buffer (0.25% casein/5 mm EDTA, pH 8.0, in PBS), including aprotinin (20 μg/ml) and leupeptin (10 μg/ml), vortexed and centrifuged at 20,000 × *g* for 20 min at 4°C. Supernatants were collected as “GuHCl fractions.” Aβ40 and Aβ42 concentrations in mouse hippocampal samples were measured using an ELISA. Briefly, rabbit polyclonal antibody specific for the C terminus of Aβ40 or Aβ42 (Millipore) was coated onto plates, and biotinylated monoclonal anti-Aβ1-16 (Covance, clone 6E10) was used for detection.

For dot blot analyses, ∼10 μg in 1 μl of lysate (TBS soluble fraction of homogenized mouse hippocampus) was blotted onto nitrocellulose membranes (#LC2001, Invitrogen) and incubated for at least 1 h at room temperature to ensure that the blots were dry. The membrane was blocked with Blocking Buffer (MB-070, Rockland Immunochemicals) with added 0.01% Tween 20, for 1 h at room temperature. The membrane was incubated with Amyloid Fibrils OC (Sigma Millipore, AB2286), Oligomer A11 (Thermo Fisher Scientific, AHB0052), 4G8 (BioLegend, 800703), or 6E10 (BioLegend, 803015) primary antibody diluted 1:1000 in Blocking Buffer for 1 h at room temperature. Total protein was normalized with mouse anti-β-actin (Cell Signaling Technology, 8H10D10, 1:10,000) or rabbit anti-GAPDH (Novus Biological, NB300-323, 1:10,000). After primary antibody incubation, membranes were washed 3 times (10 min each) with TBST (50 mm Tris, 0.5 m NaCl, 0.01% Tween 20). The membrane was incubated with secondary antibodies in Blocking Buffer at 1:15,000 dilution (IRDye 800CW donkey anti-rabbit IgG and IRDye 680LT donkey anti-mouse IgG, LI-COR Biosciences, 926-32213, 926-32212, 926-68022, and 926-68023) for 1 h at room temperature. Membrane was washed 3 times (10 min each) in TBST on rocker. Blots were scanned on Odyssey/LICOR scanner for signals followed by image analysis in Image Studio (version 5.2.5, LI-COR Biosciences).

##### CSF collection and neurofilament light chain (NfL) analysis.

A separate, mixed sex cohort of 12 month PS2APP;Trem2^wt^ (*n* = 8) and PS2APP;Trem2^ko^ mice (*n* = 8) was anesthetized, and CSF was collected from the cisterna magna and placed on ice; then blood was collected from terminal cardiac puncture, placed into EDTA collection tubes, and centrifuged at 20,000 × *g* for 2 min. (The two genotype groups in this analysis were not littermates since we had not collected plasma or CSF from our original cohorts, and we assembled this cohort just for NfL measurements due to a recommendation received during peer review.) Plasma was collected into tubes and stored at −80°C until transfer. CSF samples were diluted 1:10 in 0.1% BSA in TBS and then stored at −80°C until transfer. Plasma and CSF samples were sent to Quanterix for NfL measurements using the Simoa NF-Light Advantage Kit (product 103186). The Simoa assay is a 2 step digital immunoassay, which measures the quantity of NfL in samples using the Simoa HD-1 Analyzer and Single Molecule Array (Simoa) technology.

##### Two-photon imaging of plaque and dendritic spine measurements.

The somatosensory cortex from PS2APP mice carrying the Thy1:GFP-M transgene and different *Trem2* genotypes was imaged *ex vivo* via 2-photon microscopy. Single-sex cohorts used for this purpose included 6 month PS2APP females (*n* = 7 for per *Trem2* genotype, used for both plaque counts and dendritic spine measurements) and 8 month PS2APP males (*n* = 6 per *Trem2* genotype, used only for plaque counts). At 24 h before brain collection, animals received intraperitoneal injections of methoxy-X04 (10 mg/kg) to label amyloid structures (Klunk et al., 2002). Animals were anesthetized using isoflurane and transcardially perfused with 10 ml PBS followed by 10 ml of 4% PFA + 10% sucrose in PBS, and the collected brains were fixed overnight in 4% PFA + 10% sucrose in PBS at 4°C. After fixation, brains were mounted in agarose and immersed in PBS. Imaging and analysis were performed under blinded conditions.

Apical dendrites and their spines in somatosensory cortex upper layers were imaged *en bloc* via a two-photon laser-scanning microscope (Ultima In Vivo Multiphoton Microscopy System, Prairie Technologies) using a Ti:sapphire laser (MaiTai DeepSee Spectra Physics, Newport) tuned to 840 nm and a 60× numerical aperture 1.0 immersion objective lens (Olympus) with pixel resolution of 0.1 μm/pixel across a 1024 × 1024 pixel FOV using 1.0 μm steps, with stack depth determined by the slant of the dendritic branch being imaged. For comparison of spine density relative to plaques in PS2APP animals, an FOV containing a dendrite and nearby plaque within 20 μm was considered “near plaque” and an FOV containing only a dendrite with no visible plaque was considered “away from plaque.” To meet the “away from plaque” criteria, we confirmed that no plaque was present in the FOV and at least 100 μm outside of the containing FOV. From each brain, at least five dendrites per condition (near plaque, away from plaque) were imaged. Dendritic spine density and size measurements were generated using custom, semiautomated image analysis routines in MATLAB (The MathWorks). Spine density was estimated as the total number of visible dendritic spines divided by the corresponding length of dendrite. Relative spine volumes were estimated for each detected spine based on the number of corresponding GFP^+^ pixels in *x*, *y*, *z* dimensions above a local threshold applied as part of an automated image segmentation algorithm. For *en bloc* plaque measurements, larger-volume stacks were collected using a 20× immersion objective lens across a 1024 × 1024 pixel FOV with 2 μm steps (∼200 μm depth). Plaque density was quantified by a threshold-based MATLAB routine designed to automatically identify methoxy-X04-labeled plaques.

##### Statistical analysis.

All values are expressed as mean ± SEM. Statistical analysis was performed using the JMP (version 14.2, SAS Institute) or Prism (version 8.3.0 for Mac, GraphPad) software packages. To compare differences between PS2APP;Trem2^wt^ and PS2APP;Trem2^ko^ groups, we performed unpaired *t* tests. For comparisons of three or more groups, we performed one-way ANOVA followed by Tukey's multiple-comparisons test. The 6–7 month cohort of male and female mice was purposely analyzed separately to determine the effects of *Trem2* deficiency in each sex since female mice have accelerated amyloid pathology compared with males, and we did not want an analysis of interactions between sex and *Trem2* genotype to be confounded by the age-dependent differences in pathology between males and females.

## Results

### Plaque-associated microgliosis is impaired in PS2APP;Trem2^ko^ microglia

PS2APP mice express transgenes encoding familial AD mutations in human presenilin 2 (PS2 N141I) and APP (APP K670N/M671L). By 4 months of age, the first deposits of β-amyloid plaque are detected, with age-dependent plaque accumulation occurring faster in females than in males (Ozmen et al., 2009; Kallop et al., 2014). To determine the role of Trem2 in the progression of amyloid disease pathology, we crossed PS2APP mice with *Trem2*-deficient mice and examined plaque-related phenotypes in single-sex groups at various stages of pathology. We found a stark reduction in Iba1^+^ microglial clusters in PS2APP;Trem2^ko^ mice at 4, 6–7, 12, and 19–22 months compared with PS2APP;Trem2^wt^ (Fig. 1*A*,*B*). In addition, PS2APP;Trem2^ko^ brains showed reduced staining for CD68 marking active microglial lysosomes by IHC (Fig. 1*C*,*D*), reduced percentage of CD45^high^ (“activated”) microglia by flow cytometry (Fig. 1*E*,*F*), and reduced percentage of methoxy-X04^+^ (amyloid-containing) microglia by flow cytometry (Fig. 1*G*,*H*). Analysis of confocal *z*-stack images of cortical tissue from 12 month PS2APP;Trem2^ko^ animals also found a significant reduction in the fraction of Iba1 and methoxy-X04 signals that colocalized with each other (Fig. 1-1). We also observed reductions in total Iba1 and Gfap staining at the later ages, indicative of reduced extents of microgliosis and astrogliosis, respectively, in PS2APP;Trem2^ko^ mice (Fig. 1-1). These observations were consistent with reports of *Trem2* deletion in other β-amyloidosis models (Jay et al., 2015; Wang et al., 2015, 2016; Yuan et al., 2016; Parhizkar et al., 2019) and suggested that *Trem2* deficiency impairs the ability of microglia to engage plaques and phagocytose Aβ filbrils/aggregates.

10.1523/JNEUROSCI.1871-19.2019.f1-1 Figure 1-1 Gliosis and engagement/uptake of plaque by microglia are reduced in *Trem2*-deficient mice. A, Immunohistochemical stains for Iba1 demonstrated a significant reduction in Iba1+ area per section in *Trem2*-deficient mice. Representative Iba1 stains for the 12mo cohort are shown in Fig. 1B. B, (Top) Immunohistochemical stains for Gfap demonstrated a significant reduction in Gfap+ area per section, suggestive of reduced astrogliosis, in *Trem2*-deficient mice. (Bottom) Representative low (left, scale bar = 400 μm) and high (right, scale bar = 100 μm) magnification images of Gfap+ staining in the 12mo cohort are shown. C, Confocal z-stack images from 12mo female PS2APP cortical tissue were stained for microglia using anti-Iba1 and for plaque using methoxy-X04. Stark reductions in overlap were observed in *Trem2*-deficient mice using Manders' colocalization coefficients, corroborating the finding in Fig. 1G,H that detection of methoxy-X04+ staining was reduced in PS2APP;Trem2ko compared to PS2APP;Trem2wt microglia dissociated from brains and measured by flow cytometry. Bars and lines represent mean ± SEM. Significant differences between groups were determined by unpaired t-test, or by ANOVA followed by Tukey's multiple comparison test when more than two groups were compared (*p<0.05, **p<0.01, ***p<0.001, ****p<0.0001 versus PS2APP;Trem2wt or as indicated). Download Figure 1-1, TIF file

### Trem2-dependent induction of the proliferation and neurodegeneration-related gene expression modules

To further characterize the attenuated microglial response to β-amyloid pathology in PS2APP;Trem2^ko^ mice, we FACS-isolated the resident myeloid cell population from the cortex+hippocampus of 14–15 month PS2APP;Trem2^wt^ versus PS2APP;Trem2^ko^ mice and compared their transcriptomic profiles by RNA sequencing (raw RNA-Seq data deposited in NCBI GEO under accession number GSE140744). Although not affording single-cell resolution, our approach provided certain overall advantages (genome-wide analysis, robust detection of low-copy transcripts, and avoidance of artifactual gene expression that occurs during warm-temperature dissociations) compared with other approaches for transcription profiling of Trem2^ko^ microglia in β-amyloid models that used different cell isolation techniques and/or different RNA detection methods, such as microarray, Nanostring, or single-cell RNAseq (Wang et al., 2015; Keren-Shaul et al., 2017; Krasemann et al., 2017; Griciuc et al., 2019).

Applying cutoffs of ≥2-fold change and adjusted *p* value ≤ 0.05, we observed only 7 transcripts with increased abundance in PS2APP;Trem2^ko^ versus PS2APP;Trem2^wt^ microglia (excluding *Treml1*, an artifact of the KO cassette insertion) (Kang et al., 2018). In contrast, 144 transcripts (excluding *Trem2*) showed reduced abundance in PS2APP;Trem2^ko^ compared with PS2APP;Trem2^wt^ microglia using the same cutoffs (Fig. 2*A*; for extended data table of genome-wide expression values for each sample and summary statistics for differential gene expression, see Fig. 2-1. The majority of these transcripts showed upregulation in microglial expression profiles from the PS2APP model (Friedman et al., 2018) and other models of β-amyloid pathology (Orre et al., 2014; Wang et al., 2015) compared with nontransgenic mice (Fig. 2*A*). Therefore, their reduced expression in PS2APP;Trem2^ko^ microglia is another manifestation of the impaired microglial response to β-amyloid pathology. The dependence of these transcripts on Trem2 for their induction in PS2APP microglia was approximately concordant with published data from sorted microglial populations from the 5xFAD model (Wang et al., 2015) (Fig. 2*A*).

10.1523/JNEUROSCI.1871-19.2019.f2-1 Figure 2-1 Extended data table relating to Figure 2, with genome-wide RNA-Seq expression profiles and differential expression statistics for resident myeloid cells (Iba1+CD45+Ccr2-Ly6g-) FACS-purified from dissociated brains (cortex and hippocampus) of PS2APP;Trem2^wt^ (n=7, 5 females and 2 males) and PS2APP;Trem2^ko^ (n=6, 5 females and 1 male) at ∼14 months of age. Raw RNA-Seq data files have been deposited to NCBI GEO under accession number GSE140744. Download Figure 2-1, XLSX file

10.1523/JNEUROSCI.1871-19.2019.f2-2 Figure 2-2 Induction of genes related to proliferation and Wnt-related signaling exhibit Trem2-dependence in multiple β-amyloidosis models. **A**, Gene ontology (GO) analysis implicated several biological processes whose induction was impaired in the absence of *Trem2*, based on overrepresentation of the genes listed for a given process (false discovery rate (FDR) ≤ 0.05) among the list of 144 transcripts with at least 2-fold reduced abundance (adjusted p ≤ 0.05) in PS2APP;Trem2^ko^ vs. PS2APP;Trem2^wt^ microglia. **B**, Nine genes featured in Fig. 2B,C that had shown reduced expression in PS2APP;Trem2^ko^ microglia were analyzed in a separate dataset from independent researchers (GSE132508) for whether their expression was also reduced in Trem2^ko^ relative to Trem2^wt^ microglia, FACS-purified from 8-month 5xFAD mouse brains. Expression profiles for microglia from 8-month non-transgenic (Ntg) mice are also shown as a point of reference to visualize the extent of gene induction in 5xFAD;Trem2^wt^ versus Ntg;Trem2^wt^ microglia. Conservation of Trem2-dependence between models was observed for all except Ptk7. Bars and lines represent mean ± SEM. Significant differences between 5xFAD;Trem2^ko^ and 5xFAD;Trem2^wt^ microglia were determined by unpaired t-test (*p<0.05, **p<0.01, ****p<0.0001). Note: These statistics are not from a genome-wide analysis of differential gene expression. **C**, The Proliferation microglial gene expression module (defined in Friedman et al., 2018) was analyzed for induction in 5xFAD;Trem2wt microglia relative to Ntg;Trem2^wt^ microglia from non-transgenic mice and whether that induction was compromised in 5xFAD;Trem2^ko^ microglia. This corroborated similar findings from Fig. 2D in the PS2APP model. Lines represent mean ± SEM. Significant differences between groups were determined by ANOVA followed by Tukey's multiple comparison test (****p<0.0001 as indicated). Download Figure 2-2, TIF file

10.1523/JNEUROSCI.1871-19.2019.f2-3 Figure 2-3 Gene expression modules change in PS2APP microglia, with Neurodegeneration-related and Proliferation gene sets showing Trem2-dependence (related to Fig. 2D). The left side of these heatmaps show how the expression of several microglial gene expression modules changes in microglia FACS-purified from PS2APP versus non-transgenic (Ntg) brains, using expression data from GSE89482. The right side of the heatmaps shows whether these changes in gene expression were affected by Trem2 genotype in the current dataset. Induction of the Neurodegeneration-related and Proliferation modules was impaired in Trem2ko microglia, while the other modules were expressed to similar extents in PS2APP;Trem2^ko^ and PS2APP;Trem2^wt^ microglia. Each row represents one gene in a module, each column is one animal's microglial expression profile, and coloring represents Z-score of a sample's nRPKM value for a given gene relative to average expression for that gene across all samples within a study (not across studies, since libraries were prepared using different methods and nRPKM values between studies are not directly comparable). Overall expression scores for each gene set in each sample are plotted in Fig. 2D. Download Figure 2-3, TIF file

Four of the eight most starkly reduced transcripts (in terms of fold change) in PS2APP;Trem2^ko^ microglia are regulators of canonical Wnt signaling or proliferation (*Dkk2*, *Wif1*, *Ctnna3*, and *Asb11*) (Fig. 2*B*). Dkk2, Wif1, and Ctnna3 can all negatively regulate Wnt activity (Hsieh et al., 1999; Busby et al., 2004; Gage et al., 2008), whereas Asb11 is important for maintaining progenitor cell activity in multiple cell types (Diks et al., 2006; Tee et al., 2012). Although the lack of induction for negative Wnt regulators might suggest that Wnt-related signaling was enhanced in Trem2^ko^ microglia, another possibility is that Wnt-related signaling was impaired since Dkk2 is also a context-dependent activator of the pathway (Wu et al., 2000; Mao and Niehrs, 2003; Devotta et al., 2018) and since induction of regulators, including *Wif1*, can occur downstream of active β-catenin as negative feedback (Diep et al., 2004; Boerboom et al., 2006). Supporting this interpretation, the GO knowledge base identified “positive regulation of canonical Wnt signaling pathway” as a biological process overrepresented (fold enrichment = 10.3, FDR = 0.026) among the 144 transcripts with ≥2-fold reduced abundance in PS2APP;Trem2^ko^ microglia (Fig. 2-2*A*, with six positive factors in Wnt signaling showing reduced expression including *Fzd9* (Karasawa et al., 2002), *Sulf2* (Lai et al., 2010), *Bambi* (Lin et al., 2008), *Ptk7* (Berger et al., 2017), and *Aspm* (Buchman et al., 2011) along with *Dkk2* (Fig. 2*C*). We also analyzed recently published microglia RNA-Seq expression profiles from the 5xFAD model (Griciuc et al., 2019) and observed similar Trem2-dependent induction for the nine above-mentioned genes, with the exception of *Ptk7* (Fig. 2-2*B*. The reduced expression of Wnt-related signaling components and regulators in PS2APP;Trem2^ko^ microglia may be consistent with previous reports of coordinated signaling between Trem2 and β-catenin pathways within microglia (Zheng et al., 2017; Zulfiqar and Tanriöver, 2017).

Other GO biological processes implicated as being downregulated in PS2APP;Trem2^ko^ microglia included positive regulation of bone resorption (fold enrichment = 24.6, FDR = 0.030), protein kinase B signaling (fold enrichment = 16.2, FDR = 0.047), negative regulation of tumor necrosis factor production (fold enrichment = 14.1, FDR = 0.026), positive regulation of smooth muscle cell migration (fold enrichment = 13.9, FDR = 0.025), transmembrane receptor protein tyrosine kinase signaling pathway (fold enrichment = 5.9, FDR = 0.0037), and actin cytoskeleton reorganization (fold enrichment = 3.9, FDR = 0.044) (Fig. 2-2*A*. The Trem2-dependent genes identified in these processes may underlie described roles for Trem2 in osteoclast function (Cella et al., 2003; Paloneva et al., 2003), AKT and mTOR signaling (Ulland et al., 2017), attenuation of proinflammatory macrophage activation (Turnbull et al., 2006), chemotaxis (Mazaheri et al., 2017), and DAP12 signaling (Bouchon et al., 2001).

We recently defined a number of gene expression modules that can be used to characterize the diverse ways that microglia respond to environmental and genetic perturbations (Friedman et al., 2018). In PS2APP compared with nontransgenic microglia, several of these gene sets were upregulated, including the Neurodegeneration-related, Interferon-related, Proliferation, and LPS-related modules, whereas the Microglia and Brain Myeloid modules that typify microglia in their “homeostatic” or “resting” state were modestly but significantly downregulated (Fig. 2*D*; Fig. 2-3. The entirety of these changes is approximately equivalent to the so-called DAM (Keren-Shaul et al., 2017) or MgND (Krasemann et al., 2017) microglial activation profiles. These findings underscore the utility of these gene modules in characterizing microglial activation states. For instance, even though only 5 of 82 genes in the Proliferation module were upregulated strongly enough in PS2APP microglia to reach genome-wide significance (adjusted *p* ≤ 0.05), the overall expression of the module was clearly enriched compared with microglia from nontransgenic mice (Fig. 2*D*; Fig. 2-3.

We next analyzed the degree to which these modular changes in microglial gene expression in PS2APP mice depended on Trem2. Again, despite only 2 of 82 genes (*Ccna2* and *Aspm*) showing significant reduction in transcript abundance after correction for genome-wide analysis, the overall induction of the Proliferation module was compromised in PS2APP;Trem2^ko^ microglia (Fig. 2*D*; Fig. 2-3). We also observed less induction of the Proliferation module in Trem2^ko^ microglia expression profiles from the 5xFAD model (Griciuc et al., 2019) (Fig. 2-2*C*). The reduced expression of proliferation-related genes was consistent with the notion mentioned above that Wnt-related signaling was impaired and also corroborated reports from other β-amyloid models that proliferation markers, such as Ki67 or BrdU, were observed less frequently in *Trem2*-deficient microglia (Wang et al., 2016; Jay et al., 2017a).

As expected, the Neurodegeneration-related gene set, which overlaps with the so-called DAM (Keren-Shaul et al., 2017) and MGnD (Krasemann et al., 2017) genes but relates more specifically to neurodegenerative disease models, was notably impaired in PS2APP;Trem2^ko^ microglia (Fig. 2*D*; Fig. 2-3). Of the 134 genes in this set, 80 showed upregulation in PS2APP versus normal microglia (Friedman et al., 2018), and approximately half of these showed impaired induction in PS2APP;Trem2^ko^ microglia (adjusted *p* values ≤ 0.05) (Fig. 2*E*). Unlike the clear requirement of Trem2 for induction of many genes in the Neurodegeneration-related gene set, the downregulation of the Microglia and Brain Myeloid modules that normally occurs during any CNS challenge (Friedman et al., 2018) was not prevented by *Trem2* deletion since expression of these modules was similar in PS2APP;Trem2^wt^ and PS2APP;Trem2^ko^ microglia (Fig. 2*D*; Fig. 2-3). No effect of *Trem2* deletion on the induction of the LPS-related and Interferon-related modules was observed. Overall, our results are similar to previous analysis of the 5xFAD model, in which the microglial induction of many DAM genes showed substantial Trem2 dependence whereas the downregulation of so-called microglial “homeostatic” genes appeared largely Trem2-independent (Keren-Shaul et al., 2017).

### Plaque load is reduced in aged PS2APP;Trem2^ko^ mice

In the 5xFAD mouse model, plaque load was reportedly unchanged in 4 month Trem2^ko^ mice but increased in 8 month Trem2^ko^ hippocampus (Wang et al., 2015, 2016; Yuan et al., 2016). In the APPPS1 model, plaque load was reduced in Trem2^ko^ brains at 2 months, reduced or unchanged at 4 months, and increased in the cortex at 8 months (Jay et al., 2015, 2017a).

The reductions in microglial clustering and amyloid engulfment in PS2APP;Trem2^ko^ mice (Fig. 1) suggested that more plaque might accumulate over time in these brains, relative to PS2APP;Trem2^wt^ mice. Indeed, using the Campbell-Switzer silver stain to label amyloid plaque (Campbell et al., 1987; Switzer, 2000), we observed that plaque burden was increased in 6–7 month PS2APP;Trem2^ko^ females, and trending upward but not reaching significance in 6–7 month PS2APP;Trem2^ko^ males (Fig. 3*A*,*B*). We observed similar results in distinct cohorts of 6 month females and 8 month males by *in vivo* labeling of plaque using methoxy-X04 injection, followed by fixation and two-photon imaging of intact somatosensory cortex (Fig. 3-1). We did not observe any effect of *Trem2* deletion on the low levels of plaque deposition detected at the earliest stage examined (4 months).

10.1523/JNEUROSCI.1871-19.2019.f3-1 Figure 3-1 *Trem2* deletion increases plaque number in 6-month female somatosensory cortex. 6mo PS2APP females (**A**) or 8mo PS2APP males (**B**) with wild type (wt), knockout (ko), or heterozygous (het) *Trem2* genotypes were i.p. injected with methoxy-X04 to label brain amyloid content one day before tissue collection. Brains were perfused, fixed, and embedded in agarose for en bloc two-photon imaging of intact somatosensory cortex (∼200 μm depth) and quantitation of amyloid plaque content. Representative images are shown at left, with plots of plaque count per cubic millimeter shown to the right and each data point representing measurement from one animal. The brains used in A were the same brains used for dendritic spine measurements in Figure 8. Bars and lines represent mean ± SEM. Significant differences between groups were determined by ANOVA followed by Tukey's multiple comparison test (***p<0.001, ****p<0.0001 versus PS2APP;Trem2^wt^ or as indicated). Download Figure 3-1, TIF file

We expected to see further exacerbation of amyloid plaque load in *Trem2*-deficient brains at more advanced ages, but, to our surprise, plaque load was reduced in 12 month PS2APP;Trem2^ko^ females and in 19–22 month PS2APP;Trem2^ko^ males compared with PS2APP;Trem2^wt^ mice (Fig. 3*A*,*B*). An intermediate reduction in plaque load was observed in *Trem2* heterozygous (PS2APP;Trem2^het^) mice at 19–22 months (the only age where heterozygous mice were analyzed) (Fig. 3*A*). At this age, the Campbell-Switzer stain also revealed a distinctive pattern of “amber core” amyloid staining in PS2APP brains (Fig. 3*C*), thought to represent a mature form of highly condensed plaque. These amber cores were reduced in PS2APP;Trem2^het^ and nearly absent in PS2APP;Trem2^ko^ brains, indicating that Trem2-dependent microglial activity is essential for the formation of these particular amyloid structures.

Overall, our results are reminiscent of a recent study of APPPS1 mice, which showed that *Trem2* deletion produced increased seeding of amyloid plaques at early ages but slower rates of amyloid plaque accumulation at later ages (Parhizkar et al., 2019).

### Reduced plaque consolidation, elevated neurotoxic Aβ species, and ApoE-laden microglia in Trem2-deficient brains

**Figure 4.**
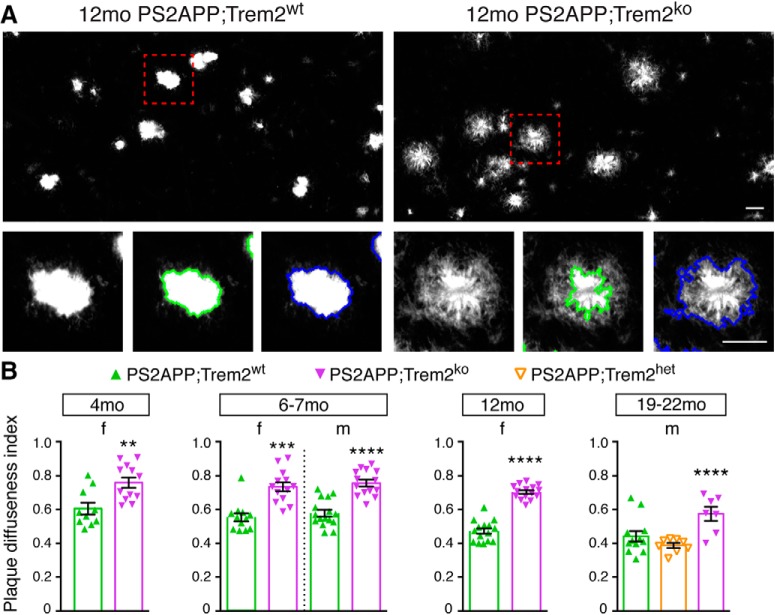
*Trem2* deletion impairs the consolidation of β-amyloid into dense plaque. ***A***, β-amyloid plaques were visualized by X-34 staining and confocal *z*-stack imaging. Representative maximum projection images of X-34^+^ plaques from 12 month PS2APP females of different *Trem2* genotypes are shown (top). High-magnification images show a representative plaque from each genotype with the outlined masks used to delineate the compact core (green) and compact+diffuse (blue) areas of the plaque (bottom). Scale bars, 20 μm. ***B***, The diffuseness index (see Materials and Methods) of the X-34^+^ plaques in cohorts of the indicated age and sex was quantified. Each data point represents 1 animal's plaque diffuseness index averaged from 3 to 4 FOV. Error bars indicate mean ± SEM. Unpaired *t* test or ANOVA followed by Tukey's multiple-comparison test: **p < 0.01, ***p < 0.001, ****p < 0.0001 versus PS2APP;Trem2^wt^.

In contrast to the effects of *Trem2* deletion on total plaque burden that varied with age or sex, we observed consistent changes in plaque compaction and composition across ages and sexes. We used the X-34 stain and confocal microscopy to visualize plaque morphology. Although we were blinded to *Trem2* genotype, there was an obvious difference in plaque appearance between PS2APP;Trem2^wt^ and PS2APP;Trem2^ko^ brains, with X-34^+^ structures in PS2APP;Trem2^ko^ brains looking more splayed and less compact (Fig. 4*A*), similar to descriptions of Trem2-dependent plaque alterations in other β-amyloid models (Wang et al., 2016; Yuan et al., 2016). Using an algorithm based on X-34 signal intensity to quantify the degree of plaque diffuseness, we observed that plaque morphologies were significantly more diffuse in PS2APP;Trem2^ko^ brains in both sexes at all ages tested (Fig. 4*B*).

**Figure 5.**
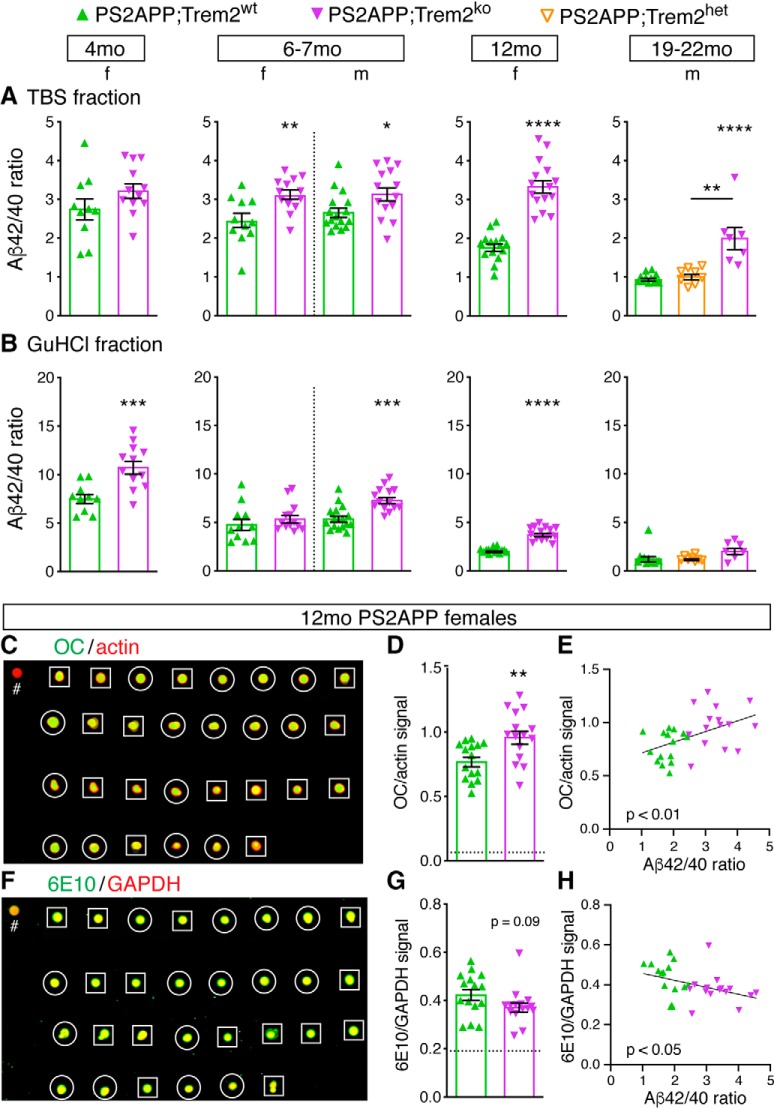
*Trem2* deletion increases the Aβ42:Aβ40 ratio and soluble fibrillar Aβ oligomers in PS2APP brains. ***A***, ***B***, Frozen hippocampal tissues were homogenized and processed for measuring the abundance of Aβ40 and Aβ42 peptides by ELISA in the soluble (TBS) and insoluble (GuHCl) fractions. The ratio of Aβ42:Aβ40 in the TBS (***A***) and GuHCl (***B***) fractions is shown. Each data point represents 1 animal's Aβ42:Aβ40 ratio. For individual Aβ40 and Aβ42 peptide measurements, see Figure 5-1. ***C***, Image of nondenaturing dot blot of hippocampal soluble TBS homogenates from 12 month female animals immunostained with the fibrillar oligomeric Aβ antibody OC (green) and control β-actin antibody (red). Squares outline PS2APP;Trem2^wt^ samples. Circles outline PS2APP;Trem2^ko^ samples. #, Control sample from a PS2APP;Bace1^ko^ mouse. ***D***, Signal intensity ratios of OC antibody to actin antibody are plotted for the dot blot shown in ***C***. Dotted line indicates the OC:actin ratio for a control sample from a PS2APP;Bace1^ko^ mouse. ***E***, The Aβ42:Aβ40 ratio (from ***A***) and normalized OC^+^ dot blot signal (from ***D***) in the TBS soluble fraction showed a significant positive correlation (linear regression; df(1,28), *F* = 8.63, *p* < 0.01). ***F***, Same as in ***C***, except immunostained with pan-Aβ antibody 6E10 (green) and control GAPDH antibody (red). ***G***, Signal intensity ratios of Aβ 6E10 antibody to control GAPDH antibody are plotted for the dot blot shown in ***F***. The PS2APP;Bace1^ko^ control sample still has substantial 6E10 signal (see dotted line) since the N-terminal Aβ residues recognized by 6E10 are present in soluble APP when α-secretase is the responsible enzyme. ***H***, The Aβ42:Aβ40 ratio (from ***A***) and normalized Aβ 6E10 dot blot signal (from ***G***) in the TBS soluble fraction showed a significant negative correlation (linear regression; df(1,28), *F* = 4.98, *p* < 0.05). Error bars indicate mean ± SEM. Unpaired *t* test or ANOVA followed by Tukey's multiple-comparison test: *p < 0.05, **p < 0.01, ***p < 0.001, ****p < 0.0001 versus PS2APP;Trem2^wt^ or as indicated.

Because stains, such as Campbell-Switzer, methoxy-X04, and X-34, only label fibrillar amyloid structures, we also measured the total abundance of Aβ40 and Aβ42 peptides in soluble (TBS) and insoluble (GuHCl) hippocampal fractions by ELISA. The abundance of Aβ peptides rose markedly with age while the Aβ42:Aβ40 ratio declined, particularly in the GuHCl fraction (Fig. 5). Notably, the Aβ42:Aβ40 ratio was higher in PS2APP;Trem2^ko^ than in PS2APP;Trem2^wt^ brains, in both TBS and GuHCl fractions across ages (Fig. 5*A*,*B*). The elevated Aβ42:Aβ40 ratio in PS2APP;Trem2^ko^ brains resulted more from reduced abundance of Aβ40 than from increased abundance of Aβ42 (Fig. 5-1), although Aβ42 abundance was elevated in the 6–7 month females, coinciding with the increased plaque deposition we observed in that group (Fig. 3*A*). Together with our observation that total amyloid plaque is reduced in PS2APP;Trem2^ko^ brains at later ages (Fig. 3*A*), these results suggest that the elevated Aβ42:Aβ40 ratio in *Trem2*-deficient brains may increase plaque seeding at younger ages (since Aβ42 is more prone to aggregate and deposit than Aβ40) (Klein, 2002) while reducing the incorporation and compaction of Aβ into existing plaques at older ages (since Aβ40 permeates dense core structures more readily than Aβ42) (Condello et al., 2015). Thus, Trem2 may both restrict the initial seeding of plaques and promote sequestration and compaction of Aβ into existing plaques.

10.1523/JNEUROSCI.1871-19.2019.f5-1 Figure 5-1 Reduced abundance of Aβ40 is more frequent than increased abundance of Aβ42 in *Trem2*-deficient PS2APP mouse brains. Frozen hippocampal tissues from cohorts of PS2APP mice with indicated age, sex, and *Trem2* genotype were homogenized in TBS, and the abundance of Aβ peptides in the TBS-soluble (A) and guanidine HCl (GuHCl)-soluble (B) fractions was measured by ELISA assays specific for detecting Aβ40 (top row) or Aβ42 (bottom row). Bars and lines represent mean ± SEM. Significant differences between groups were determined by unpaired t-test or ANOVA followed by Tukey's multiple comparison test (*p<0.05, **p<0.01, ***p<0.001, ****p<0.0001 versus PS2APP;Trem2^wt^ or as indicated). Download Figure 5-1, TIF file

To determine whether the elevated Aβ42:Aβ40 ratio was accompanied by altered abundance of soluble, fibrillar Aβ oligomers, we performed nondenaturing dot blots of hippocampal TBS homogenate supernatants from 12 month females. Using the conformation-specific OC antibody (Tomic et al., 2009), we detected significantly higher levels of soluble fibrillar Aβ oligomers in the soluble fraction from PS2APP;Trem2^ko^ mice (Fig. 5*C*,*D*), and we observed a positive correlation between the amount of OC^+^ fibrillar oligomers in this fraction and the Aβ42:Aβ40 ratio (Fig. 5*E*). In contrast, when we used the pan-reactive 6E10 Aβ antibody to detect total Aβ species in this fraction, the abundance trended slightly downward in PS2APP;Trem2^ko^ mice and correlated negatively with Aβ42:Aβ40 ratio (Fig. 5*F–H*). Similar respective trends were also seen when staining with the prefibrillar Aβ oligomer antibody A11 and pan-reactive 4G8 Aβ antibody (data not shown). As a control, we spotted hippocampal TBS homogenate supernatant from a PS2APP;Bace1^ko^ animal (Meilandt et al., 2019), in which soluble APP is still produced by α-cleavage while β-cleavage and thus Aβ production are prevented, and demonstrated that the OC antibody had minimal detection, whereas 6E10 still had substantial signal (compare # symbols in Fig. 5*C*,*F* and dotted lines in Fig. 5*D*,*G* from a PS2APP;Bace1^ko^ mouse), consistent with the ability of 6E10 to detect both soluble APP and Aβ peptides. These results suggest that, in the absence of Trem2, the increased Aβ42:Aβ40 ratio enhances the potential shift of soluble Aβ to a fibrillar oligomeric form. Alternatively, the increased abundance of fibrillar Aβ oligomers in the TBS-soluble fraction could result from reduced incorporation into highly condensed plaques in brains with *Trem2*-deficient microglia.

**Figure 6.**
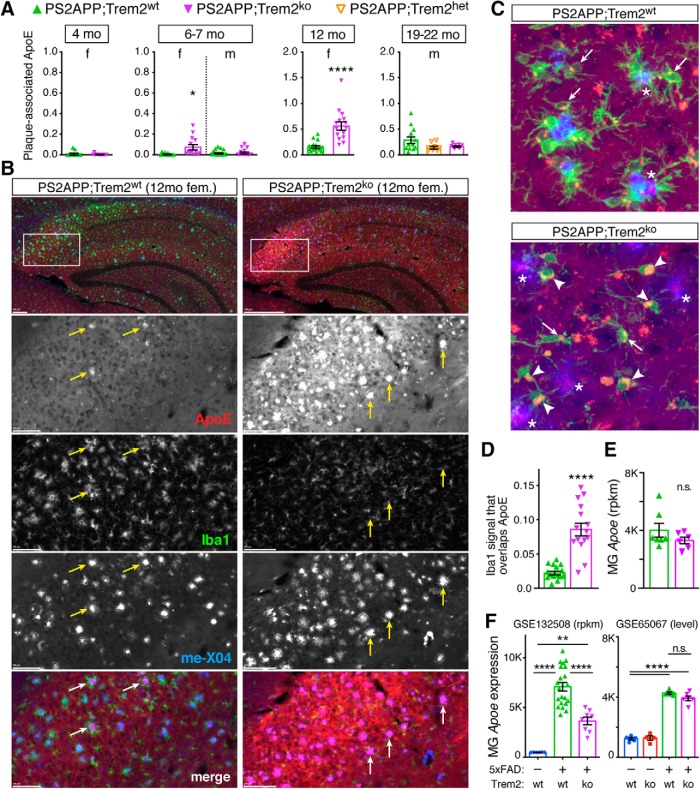
ApoE markedly accumulates on plaques and in microglia in PS2APP;Trem2^ko^ females. ***A***, Plaque-associated ApoE signal was quantified for each animal in each cohort using 2 or 3 sections per animal, with each section having a range of ∼50–800 plaques contributing to the analysis. Significant increases were observed in 6–7 month and 12 month female (f) PS2APP;Trem2^ko^ mice, but not in males (m). ***B***, Representative low-magnification images of the hippocampus (top row) and high-magnification images of the subiculum (enlarged from the boxed regions) from 12 month female brain sections stained with methoxy-X04 to label plaque (blue), anti-Iba1 to label microglia (green), and anti-ApoE (red). Arrows point to examples of plaques with intense ApoE staining, which are atypical in Trem2^wt^ females but typical in Trem2^ko^ females at this age. ***C***, Representative 20× maximum projection of confocal *z*-stacks imaged from cortex, highlighting ApoE (red) localization in microglia (Iba1, green) and plaque (blue). Microglia with small ApoE puncta (arrows) are typical in Trem2^wt^, whereas microglia with enlarged ApoE structures (arrowheads) are frequent in Trem2^ko^. Plaques with strong ApoE labeling are more common in Trem2^ko^ (asterisks). ***D***, Analysis of the fraction of Iba1 signal that overlaps with ApoE staining (Mander's colocalization coefficient) in 12 month PS2APP females revealed increased colocalization in Trem2^ko^ microglia. ***E***, RNA-Seq profiles of microglia FACS-purified from brains of 14 month PS2APP mice showed no difference in *Apoe* expression between Trem2^wt^ and Trem2^ko^ microglia (MG). ***F***, Expression profiles of microglia FACS-purified from brains of 8 month nontransgenic or 5xFAD mice revealed strong *Apoe* induction by β-amyloid pathology in both Trem2^wt^ and Trem2^ko^ microglia, with induction in 5xFAD;Trem2^ko^ relative to 5xFAD;Trem2^wt^ microglia being twofold reduced in one dataset (GSE132508, RNA-Seq) and not significantly different in another (GSE65067, microarray). Error bars indicate mean ± SEM. Unpaired *t* test or ANOVA followed by Tukey's multiple-comparison test: *p < 0.05, **p < 0.01, ****p < 0.0001 versus PS2APP;Trem2^wt^ or as indicated. n.s. - not significant.

*Apoe* is one of the most highly induced genes in mouse microglia in response to neurodegenerative stimuli (Deczkowska et al., 2018). A recent report in the APPPS1 model found that plaque-associated ApoE was reduced in *Trem2*-deficient mice (Parhizkar et al., 2019). To learn whether *Trem2* deletion altered ApoE localization in the PS2APP model and whether such alteration correlated with observed changes in plaque abundance, morphology, or composition, we costained for ApoE, plaques (methoxy-X04), and microglia (Iba1). In contrast to the findings of Parhizkar et al. (2019), quantification of plaque-associated ApoE in whole-brain sections found a significant increase in female PS2APP;Trem2^ko^ mice at 6–7 and 12 months of age, but no significant changes in male cohorts at any age tested (Fig. 6*A*). The ApoE immunostaining pattern was especially prominent in, but not limited to, the hippocampal subiculum where plaque tends to first deposit (Fig. 6*B*). While the incongruous results between studies may be explained by differences between mouse models, other variations in sampling and technical procedures could also account for the differences. Parhizkar et al. (2019) examined 4 month males; we only examined females at that age, and our findings of elevated plaque-associated ApoE in *Trem2*-deficient females were only observed at older ages. The studies used different ApoE monoclonal antibodies that likely interact with distinct epitopes or configurations of ApoE (Kim et al., 2012) and whose binding may be differentially impacted by variations in staining procedure. We immunostained for ApoE first, followed by plaque labeling, which involves treatments with ethanol and NaOH. Parhizkar et al. (2019) did the plaque labeling first, followed by the ApoE immunostains, so the nature of the ApoE at the time of immunostaining was somewhat different between studies.

Parhizkar et al. (2019) also reported that colocalization of ApoE with Iba1^+^ microglia was reduced in *Trem2*-deficient mice. To assess this finding in the PS2APP model, we performed confocal imaging on the ApoE/Iba1/methoxy-X04 costains from the cortex of 12 month females and again observed a contrasting result. Instead of ApoE labeling being diminished, we observed microglia in PS2APP;Trem2^ko^ mice to be markedly laden with ApoE (Fig. 6*C*,*D*), suggesting that *Trem2*-deficient microglia exhibit deficits in lipid clearance. At the mRNA level, we did not detect a difference in *Apoe* expression between PS2APP;Trem2^wt^ and PS2APP;Trem2^ko^ microglia (Fig. 6*E*; see also Fig. 2*E*). Although two groups have reported reductions in microglial *Apoe* expression in the APPPS1 model when *Trem2* is deleted (Krasemann et al., 2017; Parhizkar et al., 2019), this does not appear to be a typical feature of *Trem2* deficiency in β-amyloidosis models since previous analyses in the 5xFAD model observed Trem2-independent *Apoe* induction in either bulk microglia microarray (Fig. 6*F*) (Wang et al., 2015) or single-cell microglia RNA-Seq profiles (Keren-Shaul et al., 2017). In a very recent dataset of bulk microglia RNA-Seq profiles from 5xFAD mice (Griciuc et al., 2019), we did see a twofold decrease in the extent of *Apoe* induction in *Trem2*-deficient microglia, but the gene was still highly induced relative to the expression level in microglia from nontransgenic mice (Fig. 6*F*). Together, our evidence indicates that microglial *Apoe* expression is induced by Aβ-driven neuropathology in a largely Trem2-independent manner, and that *Trem2*-deficient microglia accumulate disproportionately large amounts of ApoE compared with the smaller ApoE puncta observed in normal PS2APP microglia (Fig. 6*C*).

### Axonal dystrophy, dendritic spine loss, and CSF NfL detection are exacerbated in PS2APP;Trem2^ko^ mice

The elevated Aβ42:Aβ40 ratio and fibrillar Aβ oligomers in PS2APP;Trem2^ko^ brains would seem to be detrimental for neuronal health since Aβ42 oligomers are commonly understood to be the more toxic form of Aβ (Klein, 2002; Haass and Selkoe, 2007). Alternatively, the reduced plaque load in aged Trem2^ko^ brains suggested a possible benefit of *Trem2* deficiency. Therefore, we turned to measures of neuronal dystrophy to better understand the potential consequences of loss of Trem2 function.

**Figure 7.**
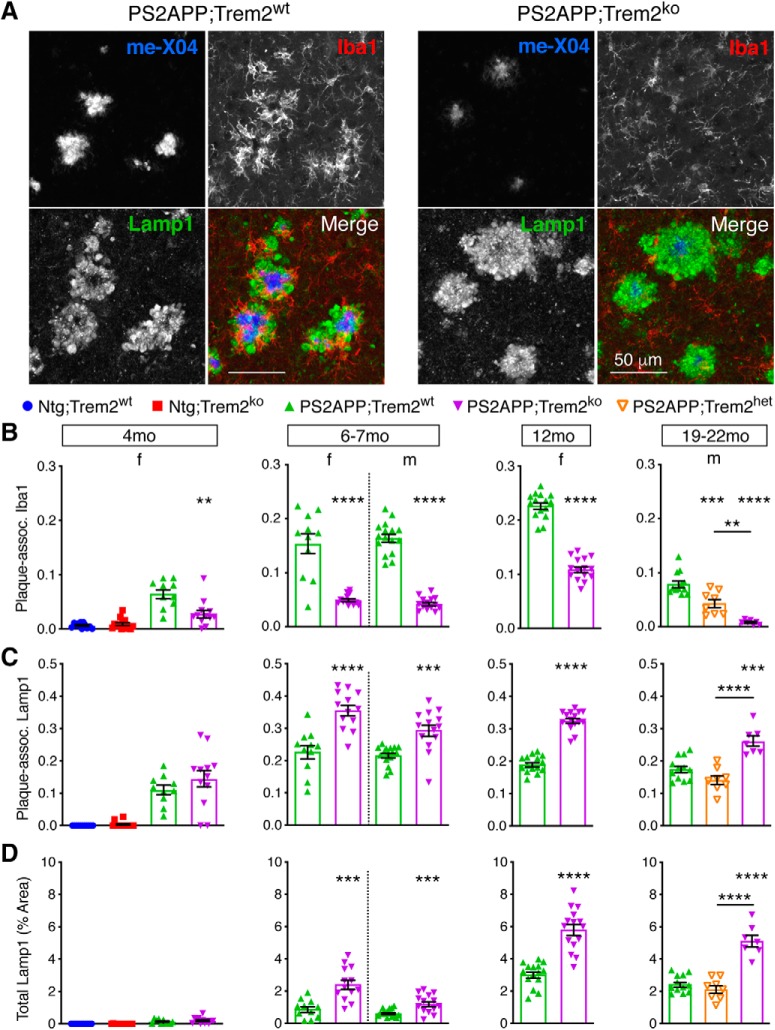
*Trem2* deletion exacerbates plaque-proximal axonal dystrophy. ***A***, Representative images from 12 month brain sections stained with methoxy-X04 to label plaque, anti-Iba1 to label microglia, and anti-Lamp1 to label dystrophic axons around plaque. ***B***, Microglial clustering around plaque is impaired in *Trem2*-deficient mice. Plaque-associated Iba1 signal was quantified for each animal in each cohort using 2 or 3 sections per animal, with each section having hundreds or thousands of plaques contributing to the analysis. ***C***, Axonal dystrophy per plaque is exacerbated in *Trem2*-deficient mice. Plaque-associated Lamp1 signal was quantified for each animal in a similar manner as Iba1 signal in ***B***. ***D***, Total axonal dystrophy is exacerbated in *Trem2*-deficient mice. Total Lamp1 signal in each section was quantified, and each data point represents the average score from 2 or 3 sections per animal. Error bars indicate mean ± SEM. Unpaired *t* test or ANOVA followed by Tukey's multiple-comparison test: **p < 0.01, ***p < 0.001, ****p < 0.0001 versus PS2APP;Trem2^wt^ or as indicated.

First, we looked at neuritic dystrophy around plaque (D'Amore et al., 2003) by fluorescent costaining using methoxy-X04 to label plaque, anti-Iba1 to label microglia, and anti-Lamp1 to label dystrophic axons (Gowrishankar et al., 2015) (Fig. 7*A*). The methoxy-X04/Iba1 costain showed that microglial association with plaque was severely compromised in PS2APP;Trem2^ko^ brains at all examined ages (Fig. 7*A*,*B*), corroborating our earlier Iba1 immunohistochemical stains that measured microglial clustering. The Lamp1 immunolabeling, which stains dilated dystrophic axons, revealed two important findings. First, on a per plaque basis, axonal dystrophy was exacerbated from 7 months onward in PS2APP;Trem2^ko^ mice (Fig. 7*C*), similar to findings in other β-amyloid models (Wang et al., 2016; Yuan et al., 2016). This is consistent with the idea that the diffuse plaque structures and elevated Aβ42:Aβ40 ratio in Trem2^ko^ brains are more damaging to surrounding axons than the more compacted, Aβ40-enriched plaques in PS2APP;Trem2^wt^ brains. Second, the total Lamp1^+^ area was also increased from 7 months onward in PS2APP;Trem2^ko^ brain sections (Fig. 7*D*), indicating that total axonal damage was exacerbated at later ages despite the reduced plaque burden. These data strongly suggest that the Trem2-dependent clustering of microglia and their functions around plaque serve to mitigate the neurotoxic effects of Aβ.

**Figure 8.**
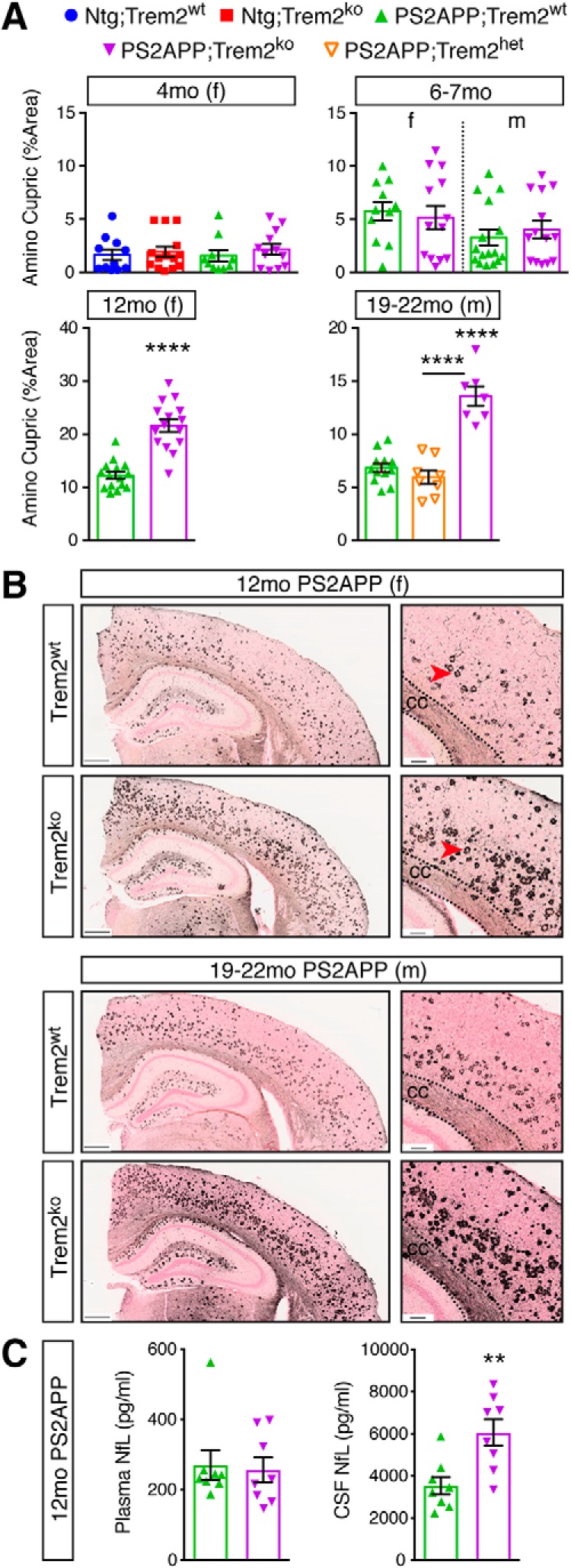
Disintegrative degeneration staining and CSF NfL measurements reveal exacerbated neuronal damage in *Trem2*-deficient mice at later ages. ***A***, Quantification of sections from nontransgenic (Ntg) and PS2APP mice with indicated *Trem2* genotypes stained using an amino cupric silver staining method that labels degenerative neuronal processes. Each data point represents the average percent area covered/section for ∼10 sections per animal. ***B***, Representative low (left, scale bar, 400 μm) and high (right, scale bar, 100 μm) magnification images from the 12 month female and 19–22 month male cohorts are shown. Degenerative signal is apparent in plaque-accompanying foci (red arrowheads) throughout the cortex and hippocampus and in certain white matter tracts, including the corpus callosum (cc). ***C***, Plasma (left) and CSF (right) NfL were measured from a separate, mixed sex cohort of 12 months PS2APP;Trem2^wt^ and PS2APP;Trem2^ko^ mice. Error bars indicate mean ± SEM. Unpaired *t* test or ANOVA followed by Tukey's multiple-comparison test: **p < 0.01, ****p < 0.0001 versus PS2APP;Trem2^wt^ or as indicated.

Another method we used to visualize neuronal pathology was an amino-cupric-silver stain or “disintegrative degeneration” stain that labels damaged or degenerating neurons (de Olmos et al., 1994; Switzer, 2000). Overall, the staining pattern appeared very similar to the Lamp1 stain, with “bouquets” of argyrophilic structures presumably surrounding plaques throughout the cortex and hippocampus. Staining was also observed in relevant white matter tracts, such as the corpus callosum, perforant path, and fornix, suggesting that degenerating axonal processes are not restricted to dystrophic neurites around plaques. Degenerating neurites detected by this stain were more abundant in PS2APP;Trem2^ko^ brains of the 12 month female and 19–22 month male cohorts (Fig. 8*A*,*B*). Thus, again, axonal damage was exacerbated in *Trem2*-deficient mice from older ages despite the fact that plaque accumulation was reduced. The intermediate effect of *Trem2* heterozygosity on microglial clustering around plaque (Figs. 1*A*, 7*B*) and Campbell-Switzer plaque staining (Fig. 3*A*), but not on plaque diffuseness (Fig. 4*B*), Aβ42:Aβ40 ratio (Fig. 5*A*), or neuritic dystrophy (Figs. 7*C*,*D*, 8*A*), suggested that the form rather than the amount of plaque correlates with neuronal damage, and that sufficient microgliosis occurs in PS2APP;Trem2^het^ mice to enable plaque compaction and neuroprotection.

NfL measured in the CSF or plasma has recently emerged as a potential biomarker of neurodegeneration in human patients and in mouse disease models (Bacioglu et al., 2016; Khalil et al., 2018). To determine whether *Trem2* deficiency altered NfL levels, we collected plasma and CSF from a separate, mixed sex cohort of 12 month PS2APP;Trem2^wt^ and PS2APP;Trem2^ko^ mice. PS2APP;Trem2^ko^ mice had significantly greater NfL levels in the CSF compared with PS2APP;Trem2^wt^ mice (Fig. 8*C*), consistent with the measures of increased axonal dystrophy that were observed by histopathology in *Trem2*-deficient females at this age. Perhaps surprisingly, we did not observe a difference in plasma NfL levels between genotypes (Fig. 8*C*), suggesting that CSF NfL measurements better represent ongoing neuronal damage or degeneration in the CNS than plasma measurements.

**Figure 9.**
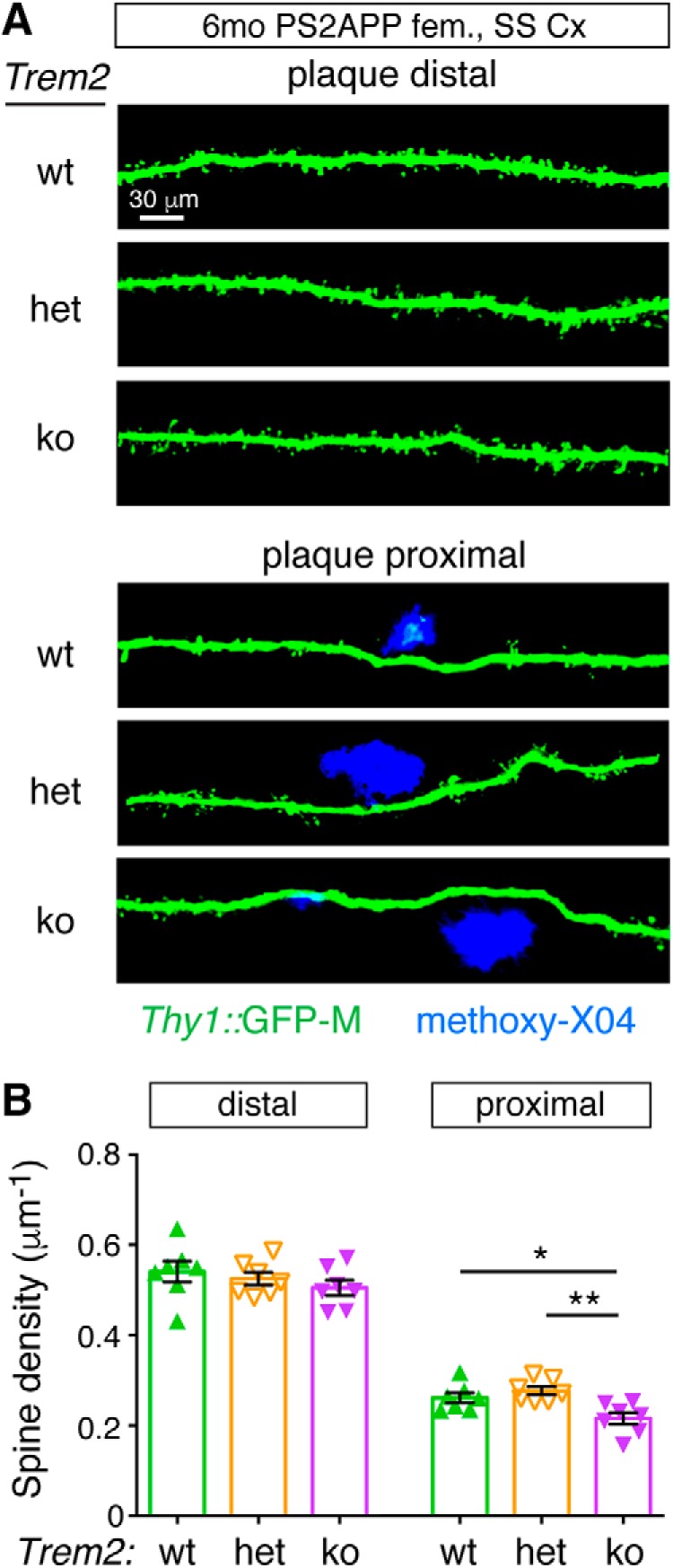
*Trem2* deletion exacerbates dendritic spine loss near plaque. ***A***, Intact brains from 6 month female PS2APP mice with WT (wt), KO (ko), or heterozygous (het) *Trem2* genotypes and carrying the Thy1::GFP-M transgene, which sparsely labels excitatory cortical neurons, were imaged in the somatosensory cortex (SS Cx) using two-photon microscopy. Dendritic shafts proximal to plaque (within 20 μm) or distally located (≥100 μm from any plaque) were imaged, with representative images shown. ***B***, Each data point represents the average of five spine density measurements from 1 animal. Spine density was ∼50% reduced on plaque-proximal dendrite segments relative to distal dendrite segments from the same animals. Spine density near plaque was lower in PS2APP;Trem2^ko^ mice than in PS2APP;Trem2^wt^ or PS2APP;Trem2^het^ mice. Error bars indicate mean ± SEM. ANOVA: *p < 0.05, **p < 0.01. Spine volume was unaffected by either plaque proximity or *Trem2* genotype (data not shown).

Finally, we looked at whether another feature of neuronal pathology observed in AD tissues and β-amyloid mouse models, reduced synaptic density, particularly near plaque (Spires and Hyman, 2004; Tsai et al., 2004; Spires et al., 2005), was altered in *Trem2*-deficient mice. In β-amyloid models, the reduction in synapse number requires the presence of microglia since depleting the microglial cell population largely prevents loss of synaptic density (Olmos-Alonso et al., 2016; Spangenberg et al., 2016). Therefore, we asked whether *Trem2* deletion and the resulting lack of activated, plaque-associated microglia would prevent the dendritic spine loss from occurring or would worsen it. To answer this, we crossed the Thy1:GFP-M line, which labels a sparse population of excitatory neurons, into the PS2APP model and analyzed a cohort of 6 month female mice with different *Trem2* genotypes. Spine density loss in the proximity of plaques was not rescued by *Trem2* deletion, but was actually further exacerbated in PS2APP;Trem2^ko^ mice compared with PS2APP;Trem2^wt^ or PS2APP;Trem2^het^ groups (Fig. 9). Thus, Trem2 is not required for microglia-mediated dendritic spine loss around plaque; and indeed, Trem2 seems to hedge against synapse loss. Overall, the exacerbated axonal dystrophy and dendritic spine loss observed around plaque in PS2APP;Trem2^ko^ mice imply that Trem2-dependent microglial activity is fundamentally neuroprotective in β-amyloid-driven models of AD-like pathology.

## Discussion

In this study, we examined the role of Trem2 in microglial activation, plaque accumulation, and neuronal dystrophy in the PS2APP model of β-amyloidosis. We observed both age- and sex-dependent effects of *Trem2* deletion on plaque abundance assessed using the Campbell-Switzer silver stain, with slightly more plaque in PS2APP;Trem2^ko^ females at 6–7 months of age but markedly less plaque in both female and male PS2APP;Trem2^ko^ mice at later ages. Together with a recent similar report (Parhizkar et al., 2019), these results suggest that Trem2-dependent microglial activity may both restrain the formation/seeding of plaques at an early stage of pathology, conceivably through uptake and degradation of soluble Aβ species, but also enhance the sequestration of Aβ into existing plaque structures, particularly at later stages of pathology.

At all ages examined, and in both sexes, microglial clustering around plaque and other measurements of microglial activation were sharply reduced in PS2APP;Trem2^ko^ mice. Transcriptional induction of the neurodegeneration-related modules, the proliferation module, and certain genes related to Wnt regulation were impaired in PS2APP;Trem2^ko^ microglia. Presumably as a consequence of the impaired microglial response around plaque, plaques in PS2APP;Trem2^ko^ brains displayed a more diffuse morphology than in PS2APP;Trem2^wt^ brains. From 6–7 months onward, axonal injury was magnified in PS2APP;Trem2^ko^ mice, even at later ages when the abundance of argyrophilic amyloid plaques was diminished. Our finding that plaque-proximal dendritic spine loss was exacerbated in PS2APP;Trem2^ko^ mice further underscored that the Trem2-dependent microglial activation around plaque is protective for neurons and is distinct from microglia-mediated, complement-mediated activities that contribute to synapse loss in β-amyloid models (Fonseca et al., 2004; Hong et al., 2016; Shi et al., 2017; Wu et al., 2019). It will be important for future studies of microglial modulation in mouse models to analyze plaque-associated neuritic dystrophy and not assume that decreases (or increases) in amyloid plaque burden are evidence of beneficial (or detrimental) effects.

While the elevated Aβ42:Aβ40 ratio we observed in the soluble fraction of PS2APP;Trem2^ko^ brains could reflect direct deficits in the Trem2-mediated binding and clearance of oligomeric Aβ42 by microglia (Yeh et al., 2016; Lessard et al., 2018; Zhao et al., 2018), it could also reflect enhanced clearance of Aβ40 through other means, such as vascular efflux. The resulting elevation in soluble Aβ42:Aβ40 ratio may give rise to the increased plaque formation we observed in 6–7 month females, similar to the increased plaque seeding activity observed by others at a similar age in another model when *Trem2* was deleted (Parhizkar et al., 2019). Given that Aβ40 is more easily incorporated into existing dense plaque structures than Aβ42 (Condello et al., 2015), our observation that the Aβ42:Aβ40 ratio declines as the mice age, particularly in the insoluble fraction, is consistent with the accumulation of compact plaques in size and number over time in the brains of PS2APP mice. The reduction of this plaque accumulation activity in PS2APP;Trem2^ko^ brains may underlie the elevated levels of fibrillar oligomeric Aβ we detected in the soluble fraction, which may in turn be a source of increased neuronal injury in these animals.

The weakness of correlation between plaque abundance and cognitive status in humans, along with several unsuccessful clinical trials aimed at preventing cognitive decline by reducing brain Aβ levels, has led some to question the relevance of Aβ in the etiology and progression of AD (Krstic and Knuesel, 2013; Morris et al., 2014; Itzhaki et al., 2016). Moreover, many have proposed that chronic microglial activation is a key damaging agent that contributes to the neurotoxic environment in AD (Heneka et al., 2015; Park et al., 2018). Our results suggest the opposite, since preventing the microglial response to Aβ pathology via *Trem2* deletion reversed neither axonal dystrophy nor dendritic spine loss around plaque and indeed made both of these pathologies worse. *Trem2* deletion also increased the amount of NfL detected in CSF. Evidence in humans and mice supports the mechanistic model that β-amyloid pathology accelerates the accumulation of tau pathology or enhances its spreading (Pooler et al., 2015; He et al., 2018; Jack et al., 2018; Jacobs et al., 2018), and a new report indicates that this effect is further magnified in *Trem2*-deficient mice (Leyns et al., 2019). Together, these findings suggest that the form of microglial activation brought on by Aβ-related pathologies (the DAM state, or at least the Trem2-dependent component of it) protects neurons by limiting Aβ-induced neuronal injury.

Supporting the notion that microglial activation is primarily beneficial in the context of AD pathology, elevated PET signal for ligands of the “neuroinflammation marker” TSPO have predicted better cognitive measures and slower AD progression in mice with β-amyloid pathology and human patients, respectively (Hamelin et al., 2016; Focke et al., 2019). The fact that plaques in Trem2-deficient mice are more injurious to adjacent neurites but show weaker labeling with molecular probes (Thioflavin S, methoxy-X04 and X-34) related to those used in the clinic (^11^C-PiB, ^18^F-Florbetapir) helps explain why cognitive decline correlates better with tau pathology and synapse loss than with brain amyloid detection. The form of β-amyloid in the brain is more critical than the amount, and Trem2-mediated microgliosis facilitates the consolidation of β-amyloid into a highly compacted, less damaging form. Therefore, therapeutics that enhance this microglial activity may prevent AD or delay its progression while simultaneously (and perhaps counterintuitively) leading to increases in PET signals for amyloid content and microgliosis. Clinical biomarkers of neuronal degeneration and spreading AD pathology, such as neurofilament-L and tau, should be more informative indicators of whether a microglia-directed therapy is achieving efficacy.
