# Supplementary figures and images for: Trem2 Deletion Reduces Late-Stage Amyloid Plaque Accumulation, Elevates the Aβ42:Aβ40 Ratio, and Exacerbates Axonal Dystrophy and Dendritic Spine Loss in the PS2APP Alzheimer's Mouse Model
Source: J Neurosci. 2020 Feb 26;40(9):1956–74. doi: 10.1523/JNEUROSCI.1871-19.2019 (PMC7046459; doi:10.1523/JNEUROSCI.1871-19.2019)

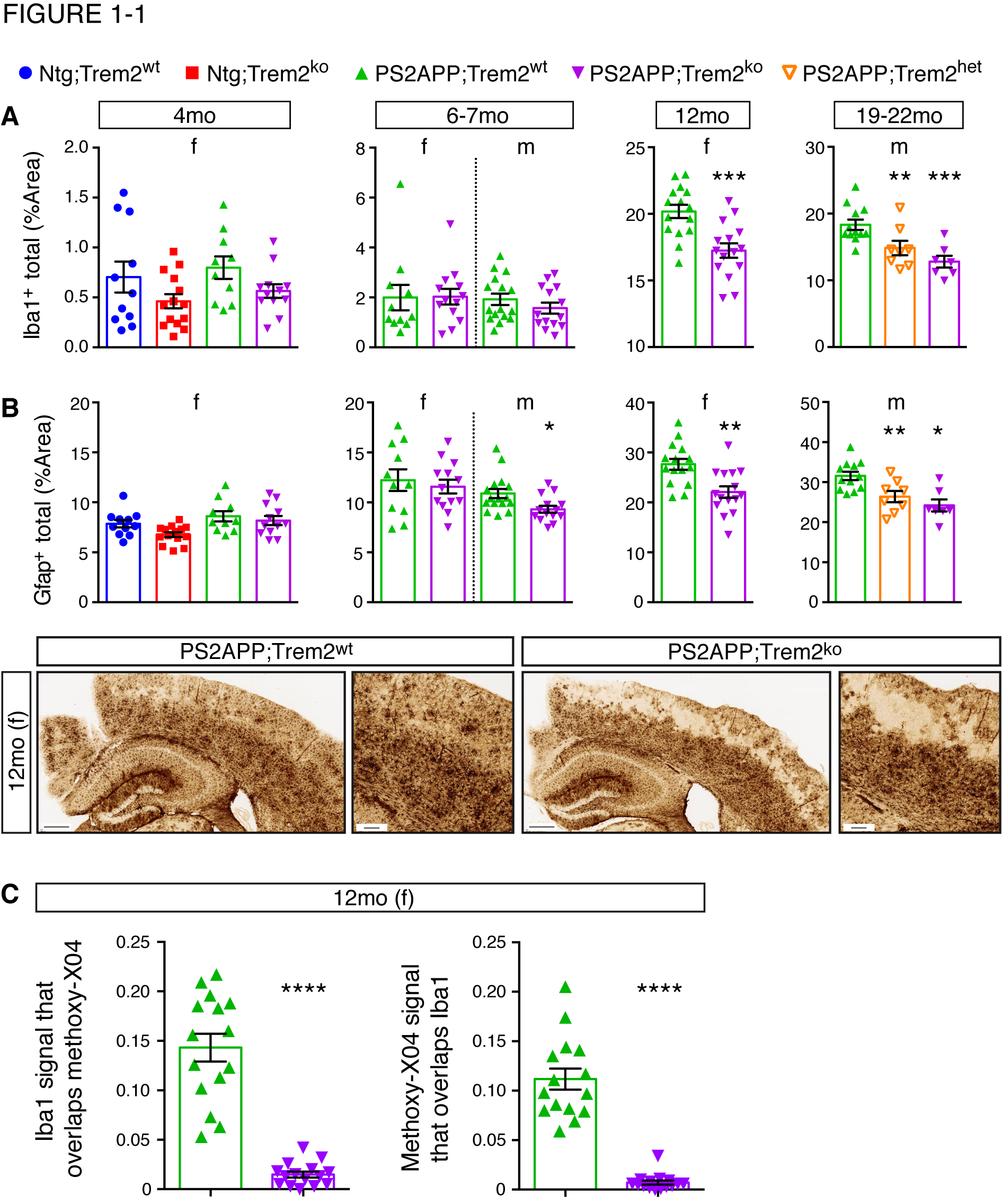

Supplement: Figure 1-1 [file sup_ns-JN-RM-1871-19-s01.tif]

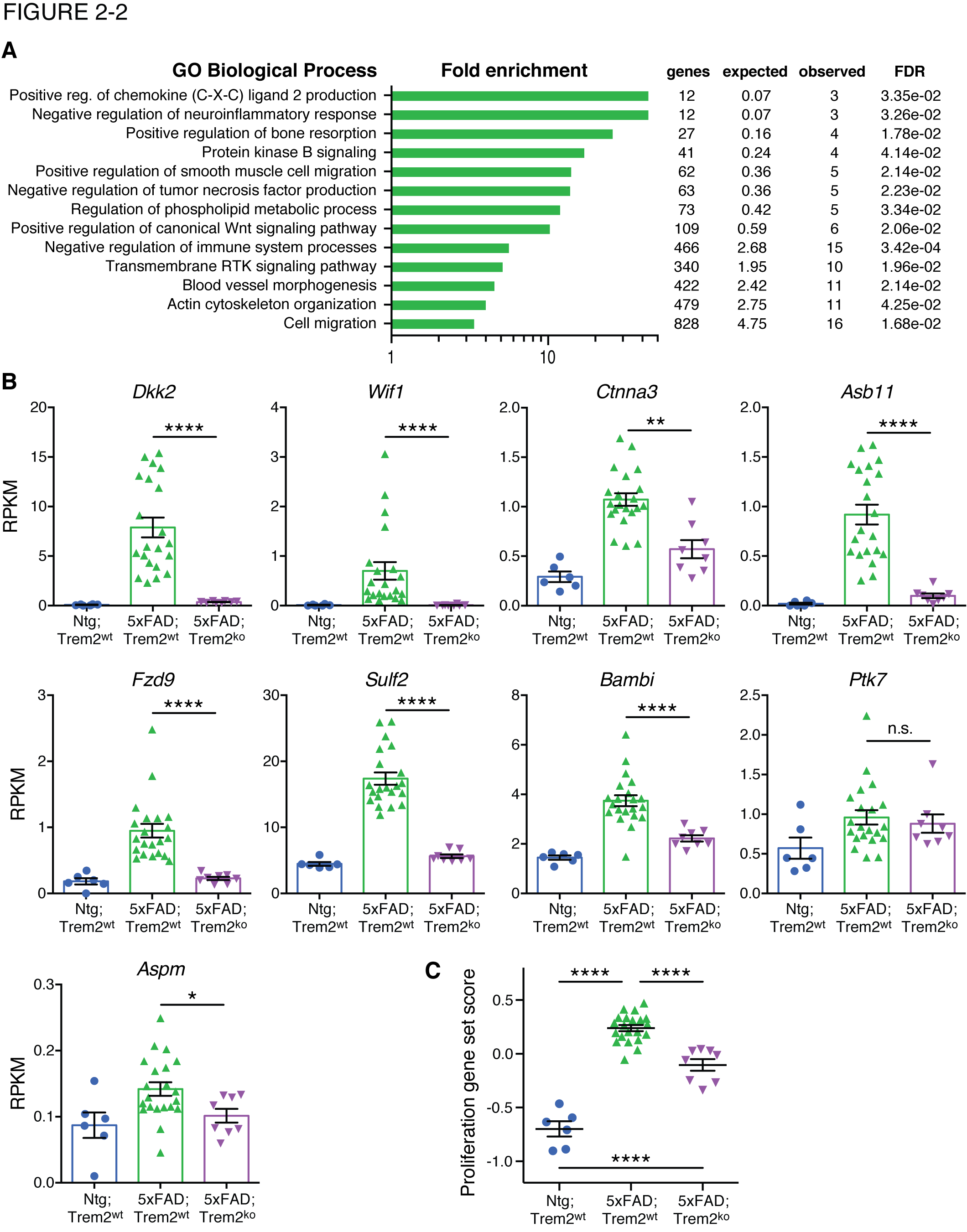

Supplement: Figure 2-2 [file sup_ns-JN-RM-1871-19-s02.tif]

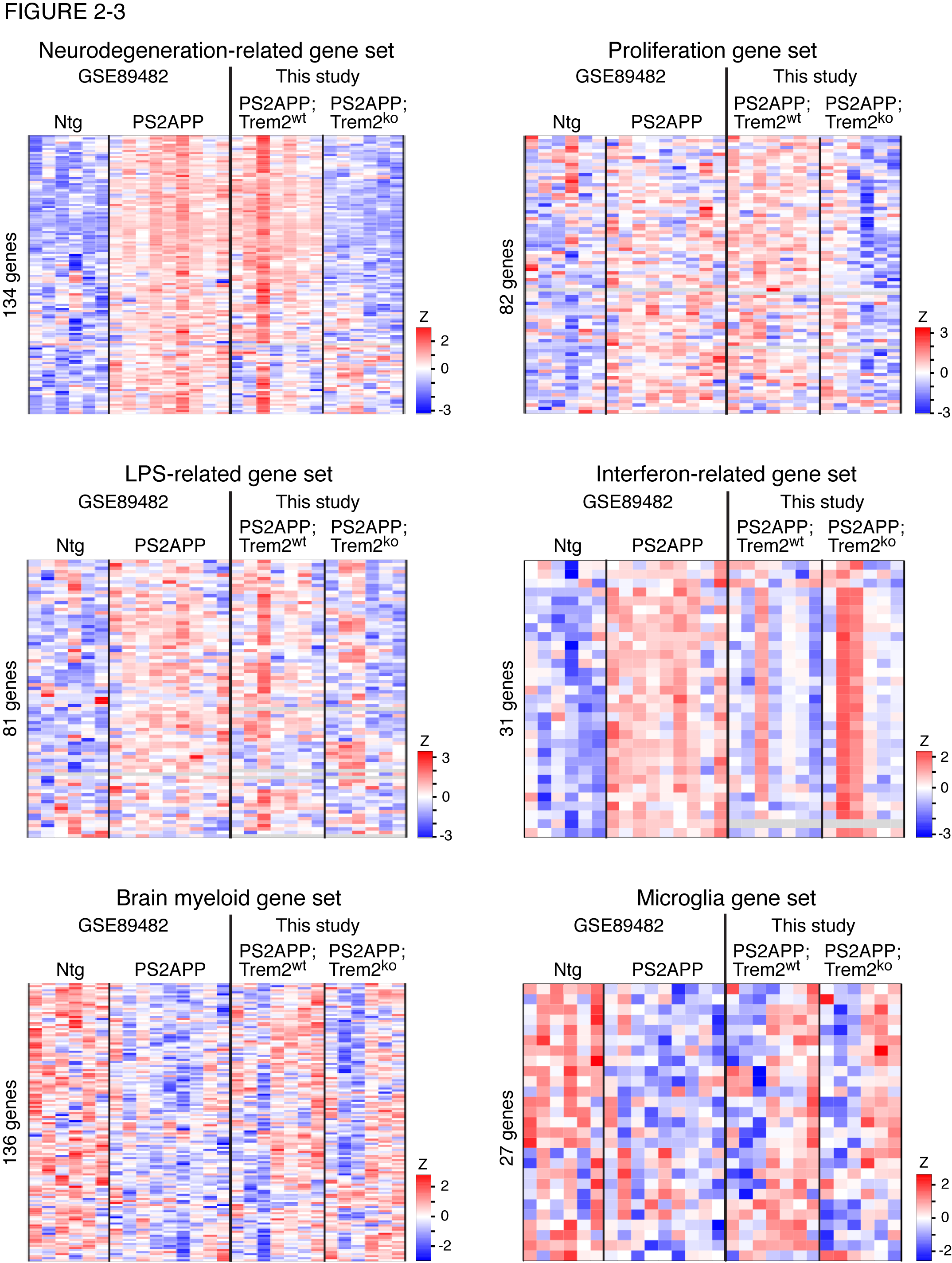

Supplement: Figure 2-3 [file sup_ns-JN-RM-1871-19-s03.tif]

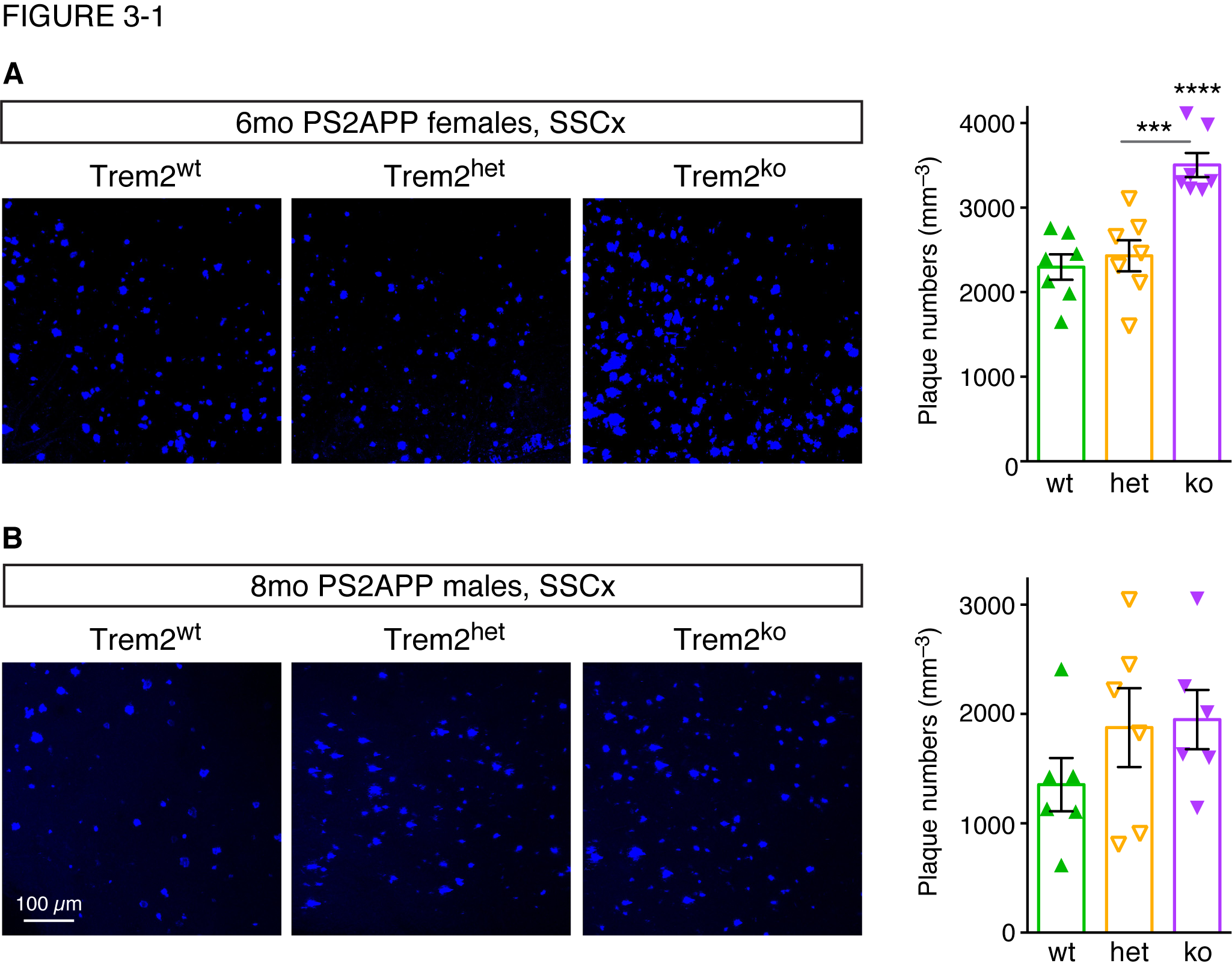

Supplement: Figure 3-1 [file sup_ns-JN-RM-1871-19-s04.tif]

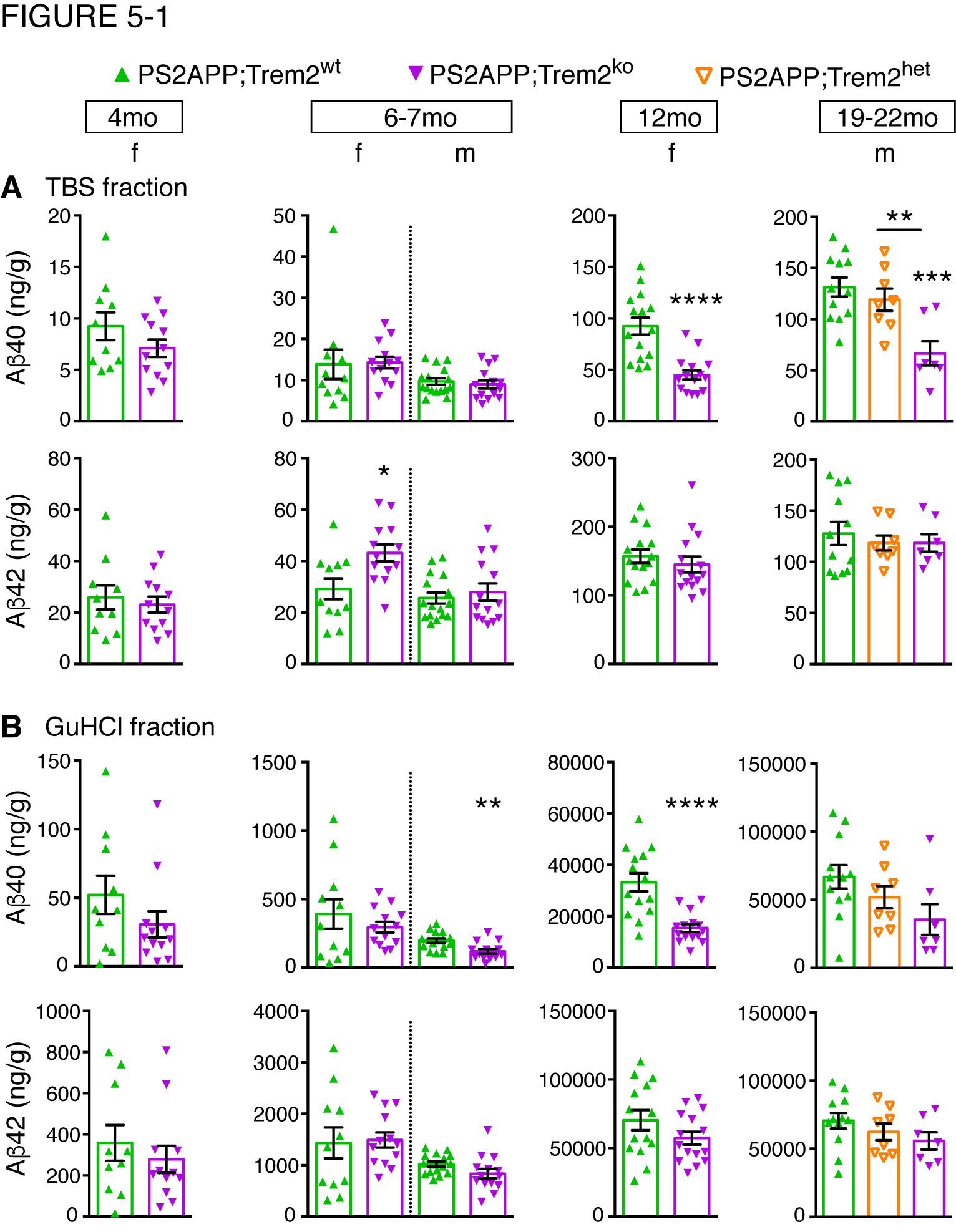

Supplement: Figure 5-1 [file sup_ns-JN-RM-1871-19-s05.tif]
